# Metabolomic Profiling Reveals PGPR-Driven Drought Tolerance in Contrasting *Brassica juncea* Genotypes

**DOI:** 10.3390/metabo15060416

**Published:** 2025-06-19

**Authors:** Asha Rani Sheoran, Nita Lakra, Baljeet Singh Saharan, Annu Luhach, Yogesh K. Ahlawat, Rosa Porcel, Jose M. Mulet, Prabhakar Singh

**Affiliations:** 1 Department of Molecular Biology & Biotechnology, College of Biotechnology, CCS Haryana Agricultural University, Hisar 125004, India; ashasheoran@hau.ac.in (A.R.S.);; 2 Department of Microbiology, College of Basic Sciences & Humanities, CCS Haryana Agricultural University, Hisar 125004, India; baljeetsaharan@hau.ac.in; 3 Department of Biotechnology, University Centre for Research and Development, Chandigarh University, Mohali 140413, India; ykahlawa@mtu.edu; 4 Allied Health Sciences, Datta Meghe Institute of Higher Education and Research, Wardha 442107, India; 5 Centre for Research Impact and Outcome, Chitkara University Institute of Engineering and Technology, Chitkara University, Rajpura 140401, India; 6 Instituto de Biología Molecular y Celular de Plantas (IBMCP), Universitat Politècnica de València-Consejo Superior de Investigaciones Científicas, Avd. de los Naranjos s/n, 46022 Valencia, Spain; 7 Department of Biotechnology, Sathyabama Institute of Science and Technology, Chennai 600119, India

**Keywords:** PGPR, Indian mustard, drought stress, primary metabolism, krebs cycle, fatty acids

## Abstract

Background: Drought stress is a major abiotic factor limiting *Brassica juncea* productivity, resulting in significant yield reductions. Plant Growth-Promoting Rhizobacteria (PGPR) have shown potential in enhancing drought tolerance; however, the metabolomic changes associated with their effects remain largely unexplored. This study examines the metabolic changes induced by a PGPR consortium (*Enterobacter hormaechei*, *Pantoea dispersa*, and *Acinetobacter* sp.) in two contrasting genotypes *B. juncea* (L.) Czern. ‘RH 725’ (drought tolerant) and *B. juncea* (L.) Czern. ‘RH-749’ (drought sensitive for drought tolerance, under both control and drought conditions. Methods: Metabolite profiling was conducted using gas chromatography-mass spectrometry (GC-MS) to identify compounds that accumulated differentially across treatments. We applied multivariate statistical methods, such as Partial Least Squares Discriminant Analysis (PLS-DA), hierarchical clustering, and pathway enrichment analysis, to explore metabolic reprogramming. Results: Drought stress induced significant changes in metabolite profile, particularly increasing the levels of osmoprotectants such as trehalose, glucose, sucrose, proline, and valine. Additionally, alterations in organic acids (malic acid and citric acid) and fatty acids (oleic acid and linoleic acid) were observed. PGPR inoculation further amplified these metabolic responses to enhance the osmotic regulation, reactive oxygen species (ROS) detoxification, and carbon-nitrogen metabolism, with RH-725 displaying a stronger adaptive response. Pathway enrichment analysis revealed that PGPR treatment significantly influenced metabolic pathways related to starch and sucrose metabolism, galactose metabolism, and amino acid biosynthesis, which play critical roles in drought adaptation. Conclusion: These findings provide insights into how PGPR contributes to stress resilience in *B. juncea* by modulating key biochemical pathways. This study provides new molecular insights into the known effect of PGPR for mitigating drought stress in oilseed crops.

## 1. Introduction

Mustard is a significant winter oilseed crop cultivated worldwide, ranking third among vegetable oil sources due to its notable medicinal, nutritional, and economic value [1,2]. *Brassica juncea* (Indian mustard) is the second most important oilseed crop after groundnut in India and is primarily grown during the rabi (winter) season across the Northern Gangetic Plains. It is also cultivated in Russia, Canada, and China. According to the Directorate of Economics and Statistics, Ministry of Agriculture and Farmers Welfare, India produced 9.12 million tons of mustard in 2020. However, despite being a leading producer, India’s mustard productivity remains low in number (about 1257 kg ha⁻^1^), significantly below the global average of 1856 kg ha⁻^1^, primarily due to various abiotic stress factors [1,3]. Among the various climatic extremes, drought stands out as a major challenge, significantly reducing agricultural productivity and economic returns. As global temperatures continue to rise, drought events are expected to become more frequent and severe [4]. This stress affects crops on multiple levels, morphological, physiological, and molecular, posing a serious threat to plant growth and yield [5]. Gaining insights into plant responses and adaptation mechanisms under drought stress is essential for devising effective strategies to mitigate its negative effects on crop productivity and ecosystem stability. Drought stress disrupts water uptake, leading to reduced seed germination. Limited moisture availability results in cellular dehydration, causing imbalances in oxidative and osmotic conditions. These disruptions alter the morpho-physiological traits of plants, hindering photosynthesis and yield-related parameters, ultimately restricting overall plant growth [6,7,8]. In *Brassica* crops, drought stress leads to a decline in leaf relative water content (RWC), chlorophyll concentration, net photosynthesis rate, and overall biomass production [9,10,11]. Additionally, drought triggers early biochemical responses that promote the generation of reactive oxygen species (ROS), including H_2_O_2_, O_2_⁻, and HO•, resulting in oxidative damage [12,13,14]. On a global scale, drought is responsible for an average annual crop yield reduction of approximately 17%, with potential losses reaching up to 70% in extreme cases [15]. The metabolomics approach has been used before to study drought stress in other *Brassicas* such as *Brassica oleracea* (Broccoli) [16,17,18].

Therefore, developing resilient crop varieties capable of withstanding abiotic stress is imperative for ensuring long-term food security [19]. While many drought mitigation strategies require substantial financial investment or the use of transgenic or edited crops, which are limited in some countries and banned for organic agriculture, emerging research highlights the potential role of microorganisms in enhancing plant tolerance to drought stress. Microorganisms are integral to soil ecosystems, playing a vital role in nutrient cycling, maintaining soil health, and enhancing plant growth through diverse mechanisms. Among them, osmotolerant microorganisms have shown promise in alleviating drought stress when applied to plants via inoculation. These beneficial microbes establish themselves in the rhizosphere, supporting plant development through both direct and indirect interactions [20]. Plant Growth-Promoting Rhizobacteria (PGPR) enhance drought tolerance by synthesizing phytohormones, producing exopolysaccharides (EPSs), and facilitating 1-aminocyclopropane-1-carboxylate (ACC) deaminase activity. They also aid in the accumulation of osmolytes and antioxidants, modulate stress-responsive gene expression, and influence root architecture to improve plant resilience under drought conditions [21,22,23,24]. Also, it may increase the biosynthesis of cytokinins [25]. Despite the well-documented role of PGPR in promoting plant stress tolerance, the underlying metabolic changes they induce remain largely unexplored, particularly in *B. juncea* under drought conditions. Metabolomics, an emerging omics approach, offers powerful insights into the biochemical mechanisms governing plant-microbe interactions. Recent studies indicate that microbial biostimulants influence plant metabolism by modulating hormonal balance, secondary metabolites, and stress-related compounds [26,27]. Key metabolites, such as sugars (trehalose, sucrose, and glucose), sugar alcohols (myo-inositol and erythritol), amino acids (proline and glycine betaine), and organic acids (malic acid and citric acid), play critical roles in osmoprotection, ROS scavenging, and metabolic homeostasis. However, the extent to which PGPR-driven metabolic adjustments contribute to drought resilience in *B. juncea* remains unclear.

This study aims to elucidate the metabolic adjustments induced by a PGPR consortium comprising *Enterobacter hormaechei*, *Pantoea dispersa*, and *Acinetobacter sp.* in two contrasting drought-responsive *B. juncea* genotypes, RH-725 (tolerant) and RH-749 (sensitive), under control and drought conditions. Genotypes ‘RH 725’ and ‘RH 749’ of *Brassica juncea* (L.) Czern. were selected from an initial screening of four genotypes (‘RH 761’, ‘RH 725’, ‘RH 30’, and ‘RH 749’) based on their contrasting responses to drought stress. ‘RH 725’ exhibited greater drought tolerance and higher yield potential, while ‘RH 749’ showed heightened sensitivity. These differences were consistently reflected in their morpho-physiological and biochemical traits under drought conditions, including relative water content, chlorophyll content, and membrane stability. Using gas chromatography-mass spectrometry (GC-MS)-based metabolomic profiling, we investigate the differential accumulation of metabolites in response to PGPR treatment. We hypothesize that PGPR treatment modulates drought-responsive metabolic pathways in *Brassica juncea* genotypes, leading to enhanced drought tolerance. This hypothesis was tested through comparative metabolomic profiling of contrasting genotypes under well-watered and drought conditions. By identifying key metabolic pathways associated with drought tolerance, this study provides insights into the biochemical mechanisms through which PGPR modulate plant metabolism, offering a foundation for developing microbial-based strategies for improving crop resilience under water-limited conditions.

## 2. Materials and Methods

### 2.1. Preparation of PGPR Consortium

A consortium of three Plant Growth-Promoting Rhizobacteria (PGPR) strains, *Enterobacter hormaechei*, *Pantoea dispersa*, and *Acinetobacter* sp., was assessed for its efficacy. The consortium was prepared in a 1:1:1 ratio. Bacterial suspensions were generated by centrifuging bacterial cultures grown to an OD_600_ of 1.0 (10^8^ CFU/mL) at 4000 rpm (~3000× *g*) for 20 min using an Eppendorf 5810R refrigerated centrifuge. After which the cell pellets were resuspended in sterile distilled water [28,29].

### 2.2. Seed Preparation and Inoculation

Seeds of *Brassica juncea* varieties RH-725 and RH-749 were surface-sterilized using 0.1% HgCl_2_ for one minute, followed by three rinses with sterile distilled water, coated with a 15% gum arabica solution, and soaked in the bacterial suspension for six hours to ensure full contact between the seeds and bacterial cells [28,29]. The control seeds were soaked in sterile distilled water.

### 2.3. Experimental Setup and Growth Conditions

Seeds obtained from the Oilseed Section Department of Genetics and Plant Breeding, CCSHAU, were sown (October sowing) in earthen pots (frustum-shaped: 25.5 cm height, 26 cm top diameter, and 18.5 cm bottom diameter), in a greenhouse at the College of Biotechnology, CCSHAU, Hisar. The pots were filled with soil (~7 kg), saturated with tap water (pH~7.5), and allowed to drain overnight to determine field capacity (~2000 mL). After three days, the six seeds were sown per pot, and thinning was performed on three seedlings per pot after germination. The plants were watered regularly for 60 days under controlled conditions (light- 75 W/m^2^, 65% RH, and temp 25 *±* 2 °C). After 60 days, the pots were divided into two groups: one was watered consistently (~300 mL every 2nd day) as a control, while the other group was subjected to drought stress by withholding water for 10 days (field capacity ~25%) when wilting was observed. So, it makes a total of four treatments: Control (regularly watered), PGPR treated (regularly watered), Drought, and Drought + PGPR. All treatments were conducted in biological triplicate; samples were collected and stored at −80 °C.

### 2.4. Sample Preparation and Derivatization

Leaf and root samples were ground into a fine powder using liquid nitrogen. Leaf tissue (excluding the midrib) and root segments from ~3 cm from the tip of the primary root were used for metabolite extraction. To inactivate enzymes, 700 μL of methanol (precooled to −20 °C) was added to 150 mg of the plant sample in a 2 mL Eppendorf tube, which contained 60 μL of ribitol (0.4 mg/mL stock in milli-Q water) as an internal quantitative standard. The tube was vortexed for 10 s and then centrifuged at 11,000× *g* for 15 min. The supernatant was transferred to a new 2 mL Eppendorf tube, and 700 μL of milli-Q water and 370 μL of chloroform were added. The mixture was vortexed again and centrifuged at 2200× *g* for 10 min. Aliquots of the polar layer were dried in a vacuum concentrator, and the dried extract was stored at −20 °C until analysis. For derivatization, 40 μL of methoxyamine hydrochloride in pyridine (20 mg/mL) was added to the dried samples. The mixture was vortexed and incubated on a thermomixer at 30 °C for 1.5 h. Subsequently, 70 μL of MSTFA was added to convert the organic acids into volatile trimethylsilyl derivatives, and the mixture was incubated for 30 min at 30 °C [30].

### 2.5. GC–MS Analysis

GC–MS analysis was carried out using a 7890A gas chromatograph (Agilent Technologies, Santa Clara, CA, USA), equipped with an Agilent Technology GC autosampler 120 (PAL-LHX-AG12). Helium served as the carrier gas at a flow rate of 1 mL/min, and samples were injected in split mode with an injection temperature of 230 °C. Ribitol was used as a quality control standard before and after each batch, and its retention time was recorded for consistency. A blank was run between samples to prevent contamination. The endpoint was defined based on the standard total ion chromatogram (TIC) time window for GC-MS runs. Electron ionization at 70 eV was used as the ionization source for GC–MS analysis, and data acquisition was performed in full scan mode (*m/z* 50–600) to allow for untargeted metabolite profiling and blank verification.

### 2.6. Metabolite Data Analysis

Metabolite peak data under control, PGPR consortium, drought stress, and drought stress with PGPR consortium treatments were formatted as comma-separated value (.csv) files and uploaded to the MetaboAnalyst 6.0 server (http://www.metaboanalyst.ca) accessed on 11 June 2024. To minimize potential variance and enhance the accuracy of subsequent statistical analyses, the data were assessed for integrity and normalized using MetaboAnalyst’s built-in protocols. Normalization was performed through sum normalization, log transformation, and auto-scaling to ensure optimal performance in statistical analysis. Univariate analysis, including *t*-tests and one-way ANOVAs, was conducted to assess the statistical significance of metabolites between group means (Drought vs. Control, PGPR vs. Control, and PGPR + Drought vs. Drought). Metabolites with a fold change greater than 2 and a *p*-value below 0.05 were considered statistically significant. Fold change values were obtained using the Fold Change Analysis module in MetaboAnalyst 6.0. The fold change represents the ratio of average metabolite abundance in different treatments. While fold change values themselves do not include error margins, statistical significance was determined using univariate analysis (*t*-test or ANOVA), and corresponding *p*-values are provided to indicate variability across biological replicates. Since multivariate methods account for all variables collectively, they were employed for a more comprehensive data analysis. These included supervised techniques such as Partial Least Squares Discriminant Analysis (PLS-DA), along with hierarchical clustering with heatmaps. Heatmaps were constructed using Pearson distance measurements and the Ward clustering algorithm [31]. The connection between metabolic pathways and associated metabolites was analyzed using MetaboAnalyst.

## 3. Results

Wilting symptoms were first observed in RH-749 after 10 days without irrigation. At the time of sampling, leaf relative water content (RWC) was 59.85 ± 3.61% in RH-725 and 40.27 ± 0.88% in RH-749, confirming significant genotypic variation in drought response.

### 3.1. Metabolome Profiling

Differential accumulation of metabolites was observed in RH-725 and RH-749 roots and leaves by application of PGPR under control and drought conditions. The representative chromatograms for each treatment have been provided in the Supplementary File S1.

A total of 167 metabolites were detected across all samples. Among these, amino acids, amines, organic acids, sugars, sugar acids, sugar alcohols, and fatty acids were categorized as major groups based on their relative abundance and well-established roles in plant drought stress response. Only the metabolites having a fold change of more than 2 across different treatment groups and a *p* value less than 0.05 were considered differentially abundant.

#### 3.1.1. Effects of Drought Stress on Metabolite Profile

Drought stress induced substantial metabolic reprogramming in both genotypes, although the extent of these changes varied between RH-725 and RH-749.

##### Metabolite Changes in RH-725 Under Drought Stress

In RH-725 leaves, 10 metabolites showed upregulation while 6 exhibited downregulation under drought stress. Notable increases were observed in several metabolite categories. Sugars, trehalose (42.66-fold) and amino acids such as proline (35.55-fold) and valine (38.04-fold) showed significant increases (Table 1). Downregulation was observed in several categories, including sugars such as fructose (16.88-fold), amino acids such as ethanolamine (2.17-fold), L-5-oxoproline (2.29-fold), and other notable metabolites include ribono-1,4-lactone (2.17-fold), urea (2.27-fold), and stigmast-5-ene (2.35-fold) (Table 2).

In the roots of RH-725, 10 metabolites were upregulated and 12 were downregulated in response to drought stress. Notable increases were observed in sugars such as melibiose (40.22-fold) and glucose (23.86-fold). The amino acids glycine (3.39-fold), proline (2.97-fold), and valine (4.21-fold) also showed significant upregulation. Among organic acids, malic acid (2.28-fold) displayed increased levels (Table 1). On the other hand, significant decreases were noted in sugars, including methyl galactoside (8.91-fold) and turanose (2.14-fold), amino acids ethanolamine (2.03-fold), L-5-oxoproline (2.03-fold), and serine (2.21-fold). Organic acids, such as butanedioic acid (2.14-fold) and galactonic acid (2.97-fold), also showed decreased levels. Additionally, oleic acid (6.93-fold) and ribono-1,4-lactone (2.37-fold) exhibited significant downregulation (Table 2).

##### Metabolite Changes in RH-749 Under Drought Stress

In the leaves of RH-749, drought stress resulted in the upregulation of 4 metabolites and the downregulation of 10 metabolites. Among amino acids, proline (24.68-fold) and glutamic acid (3.39-fold), and among the sugars, talose (7.08-fold) showed significant increases. Myo-inositol (9.04-fold) was another significantly elevated metabolite (Table 1). Conversely, significant reductions were observed in metabolites such as propanetricarboxylic acid (6.43-fold), ribono-1,4-lactone (5.32-fold), stearic acid (5.79-fold), bis(2-ethylhexyl) phthalate (5.59-fold), and cellobiose (2.98-fold decrease) (Table 2).

In the roots of RH-749 under drought stress, 7 metabolites were upregulated while 6 metabolites were downregulated. Significant increases were observed in sugars such as arabinose (5.19-fold) and trehalose (6.00-fold). The amino acids aminobutanoic acid (2.38-fold) and glutamic acid (2.97-fold) were notably upregulated. Oleic acid (5.01-fold) and Myo-inositol (26.53-fold) showed elevated levels (Table 1). Notable decreases included meso-erythritol (361.58-fold), sorbose (2.44-fold), silanol (2.11-fold), and threonic acid (2.62-fold) were observed (Table 2).

**Table 2:** Metabolites downregulated in response to drought stress.

Sample Name	Metabolite Name	Fold Change (Control/ Drought)	log2 (Fold Change)	Raw. *p* Val	Class
RH-725 Leaves	Fructose	16.881	4.0773	0.017021	Sugar
	Stigmast-5-ene	2.351	1.2333	0.02766	Other (Phytosterol)
	L-5-Oxoproline	2.2886	1.1945	0.010638	Amino acid
	Urea	2.2713	1.1835	0.031915	Other (Amide)
	Ribono-1,4-lactone	2.1726	1.1194	0.012766	Other (Lactone)
	Ethanolamine	2.1664	1.1153	0.048936	Amine
RH-725 Roots	Methyl galactoside	8.9131	3.1559	0.034884	Sugar
	Oleic acid	6.933	2.7935	0.015163	Fatty acid
	Pentanedioic acid	6.2312	2.6395	0.023256	Organic acid
	SILANOL	5.2106	2.3814	0.004651	Other
	Galactonic acid	2.9734	1.5721	0.062791	Sugar acid
	Talose	2.5905	1.3732	0.018605	Sugar
	Ribono-1,4-lactone	2.3741	1.2474	0.005116	Other (Lactone)
	Serine	2.2148	1.1472	0.030233	Amino acid
	Turanose	2.1416	1.0987	0.027907	Sugar
	Butanedioic acid	2.1364	1.0952	0.016279	Organic acid
	L-5-Oxoproline	2.0348	1.0249	0.04186	Amino acid
	Ethanolamine	2.0278	1.0199	0.037209	Amine
RH-749 Leaves	meso-Erythritol	361.58	8.4982	0.037037	Sugar alcohol
	Stearic acid	5.7936	2.5345	0.025926	Organic acid
	Bis(2-ethylhexyl) phthalate	5.5863	2.4819	0.040741	Other
	Scyllo-Inositol	5.5495	2.4724	0.011111	Sugar alcohol
	Acetin	4.7995	2.2629	0.003704	Other (Ester)
	Cellobiose	2.976	1.5734	0.033333	Sugar
	6,7-DIHYDROXYCOUMARIN	2.9244	1.5481	0.018519	Other
	Arabinose	2.8182	1.4948	0.044444	Sugar
	Stigmast-5-ene	2.5413	1.3456	0.015852	Other (Phytosterol)
	INOSITOL	2.4784	1.3094	0.007407	Sugar alcohol
RH-749 Roots	PROPANETRICARBOXYLIC ACID	6.429	2.6846	0.004255	Organic acid
	6,7-DIHYDROXYCOUMARIN	5.4608	2.4491	0.02766	Other
	Ribono-1,4-lactone	5.3218	2.4119	0.031915	Other (Lactone)
	Threonic acid	2.6216	1.3904	0.021277	Sugar acid
	Sorbose	2.4383	1.2859	0.029787	Sugar
	SILANOL	2.1086	1.0763	0.023404	Other

#### 3.1.2. Metabolomic Adjustments Induced by PGPR Under Control and Drought Conditions

PGPR application significantly influenced metabolite profiles in both genotypes, with differential effects observed under control and drought conditions.

##### PGPR-Induced Changes in Metabolites Under Control Conditions

In the leaves of RH-725, 17 metabolites were upregulated and 6 downregulated in response to PGPR application under control conditions. Significant increases were observed in fructose (43.89-fold) and glucose (10.70-fold). Among amino acids, glycine (5.12-fold) and proline (4.81-fold) were significantly upregulated. Additionally, there was an upregulation of myo-inositol (3.33-fold) (Table 3). Fructose showed a notable decrease (9.27-fold), along with gluconic acid (7.61-fold) and quininic acid (4.11-fold) (Table 4).

In RH-749 leaves, 10 metabolites were upregulated and 9 were downregulated under control conditions. Notable increases were observed in proline (16.61-fold) and glutamic acid (5.93-fold), alongside sugars like talose (12.61-fold), mannobiose (7.27-fold), and trehalose (7.15-fold). The sugar alcohol myo-inositol was significantly upregulated (8.13-fold), as was glycerol (5.05-fold) (Table 3). Butanedioic acid showed a notable decrease (18.50-fold), along with malic acid (105.64-fold) and linoleic acid (2.60-fold) (Table 4).

In RH-725 roots, 10 metabolites were upregulated, and 9 were downregulated in response to PGPR application under control conditions. Significant increases were observed in galactose (16.96-fold), xylose (7.67-fold), and mannobiose (6.29-fold). The amino acid proline showed a substantial increase (8.00-fold). Additionally, uridine (11.03-fold), gluconic acid (4.58-fold), and aminobutanoic acid (2.05-fold) were notably elevated (Table 5). Significant decreases were observed for inositol (2.51-fold), isoleucine (3.75-fold), dihydroxybutanoic acid (3.25-fold), pentanedioic acid (2.81-fold), and propanedioic acid (2.05-fold) (Table 6).

In RH-749 roots, 7 metabolites were upregulated and 4 were downregulated in response to PGPR under control conditions. Significant upregulation was observed for myo-inositol (28.93-fold) and oleic acid (5.33-fold). Trehalose showed a notable increase (3.52-fold), along with ribose (2.01-fold) and threonine (2.28-fold) (Table 5). Notable decreases were observed for gluconic acid (8.61-fold), glycine (3.67-fold), and pentanedioic acid (2.06-fold) (Table 6).

##### PGPR-Induced Changes in Metabolites Under Drought Stress

Under drought conditions, 11 metabolites were upregulated while 9 were downregulated in RH-725 leaves by PGPR application. Significant upregulation was observed in fructose (43.89-fold), glucose (10.74-fold), glycero-D-gulo-heptose (4.52-fold), and galactose (4.36-fold) alongside other metabolites such as oleic acid (3.69-fold) and threitol (10.74-fold) (Table 3). Significant downregulation was observed in quininic acid (173.71-fold) and sucrose (77.26-fold). Aspartic acid showed a substantial decrease (4.65-fold), with myo-inositol (15.99-fold). Other notable decreases included threonine (2.64-fold) and tromethamine (5.39-fold) (Table 4).

Under drought conditions with PGPR application, 5 metabolites were upregulated and 5 were downregulated in RH-749 leaves. Significant upregulation was observed in arabinose (7.87-fold), galactose (3.69-fold), and cellobiose (2.18-fold) (Table 3). Significant downregulation was observed for gluconic acid (8.61-fold), pentanedioic acid (2.06-fold), and glycine (3.67-fold) (Table 4).

In RH-725 roots, 8 metabolites were upregulated and 7 were downregulated in response to PGPR application under drought stress. Significant increases were observed in sugars like psicose (14.61-fold) and galactose (3.22-fold). Proline was significantly upregulated (8.00-fold), along with lanthionine (2.13-fold) (Table 5). Notable decreases were observed in malic acid (390.29-fold), valine (25.54-fold), arabinonic acid (9.02-fold), butenedioic acid (4.51-fold), and sucrose (8.10-fold). Threonine (14.75-fold) and inositol (4.02-fold) also exhibited significant downregulation (Table 6). RH-749 roots exhibited 6 metabolites upregulated and 8 downregulated in response to PGPR application under drought stress. Significant increases were observed in sugars such as tagatose (7.74-fold), sucrose (2.99-fold), and turanose (3.73-fold). Proline was notably upregulated (2.71-fold) along with threonic acid (2.28-fold) (Table 5). Significant decreases were observed in stearic acid (9.54-fold), arabinonic acid (2.58-fold), and aminobutanoic acid (2.15-fold). Glutamic acid (3.58-fold), ribonic acid (3.70-fold), and inositol (2.50-fold) were also significantly downregulated (Table 6).

### 3.2. Multivariate Analysis of Metabolite Profiles

Partial Least Squares Discriminant Analysis (PLS-DA) was used to visualize variations in metabolite accumulation across different treatments.

**Figure 1:**
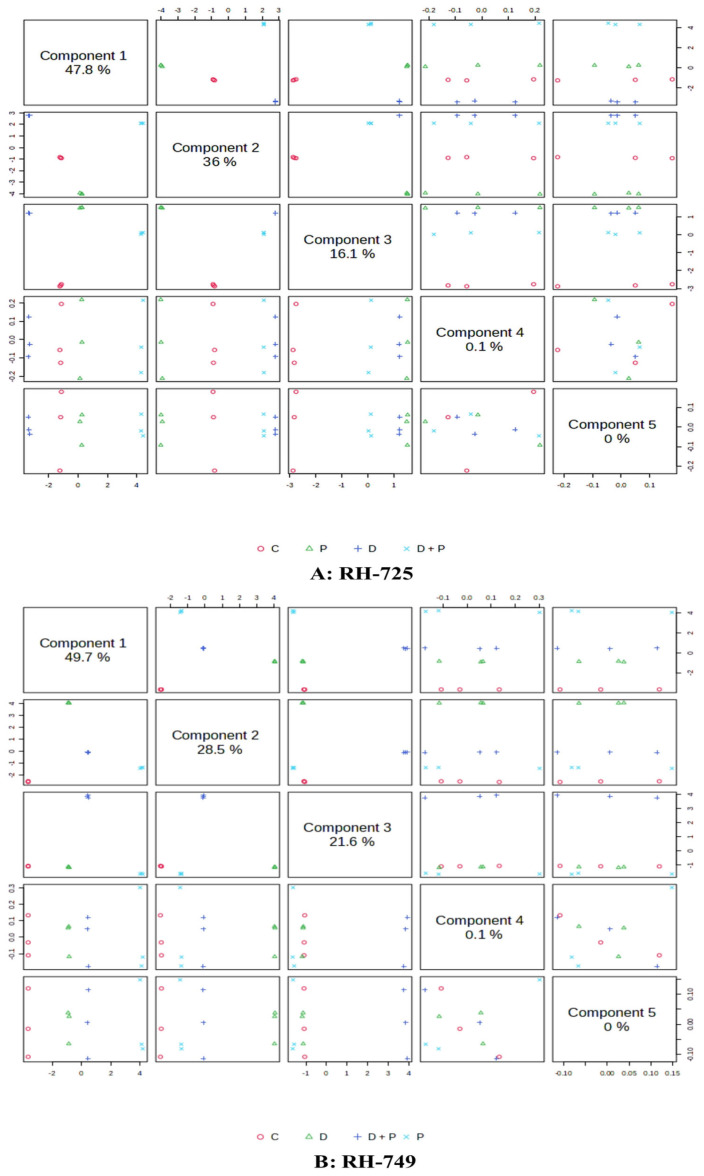
Partial least squares discriminant analysis (PLS-DA) score plots depicting variation in metabolites accumulated in leaves of *Brassica juncea* genotypes (**A**: RH-725, **B**: RH-749) under treatments (C: Control, P: PGPR, D: Drought, and D + P: Drought + PGPR). Metabolite data were derived from GC-MS. PLS components represent the major axes of variation, highlighting treatment-induced metabolic shift.

**Figure 2:**
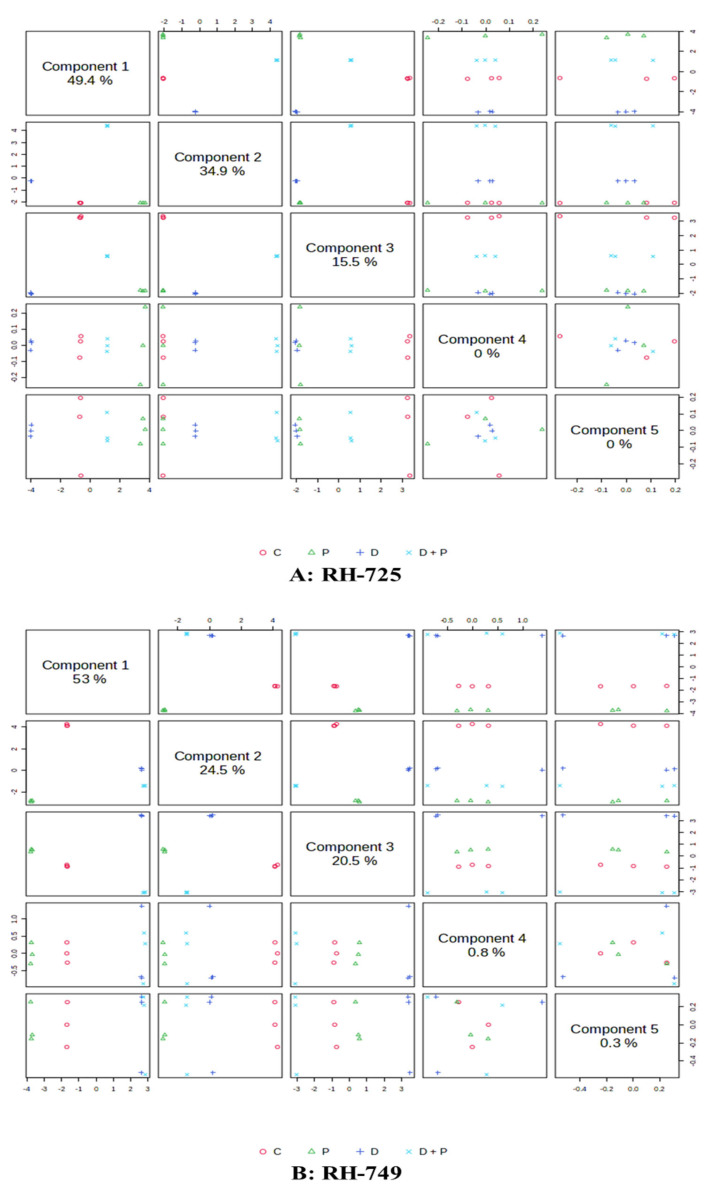
Partial least squares discriminant analysis (PLS-DA) score plots depicting variation in metabolites accumulated in roots of *Brassica juncea* genotypes (**A**: RH-725, **B**: RH-749) under treatments (C: Control, P: PGPR, D: Drought, and D + P: Drought + PGPR). Metabolite data were derived from GC-MS. PLS components represent the major axes of variation, highlighting treatment-induced metabolic shift.

PLS-components (PCs) analysis of leaf metabolites showed that the first component explained 47.8% and 49.7% of the total variation in RH-725 and RH-749, respectively, when comparing PGPR-treated plants to non-treated ones. The second component accounted for 36.0% and 28.5% of the variation in RH-725 and RH-749 leaves, respectively, under the same treatment (Figure 1). PLS-components (PCs) analysis of roots metabolites revealed that component 1 explained 49.4% and 53.0% of the total variation of the RH-725 and RH-749 roots, respectively, under PGPR versus non treated plants; the second component explained 34.9% and 24.5% of the variation for RH-725 and RH-749 roots, respectively, for the same treatment (Figure 2).

**Figure 3:**
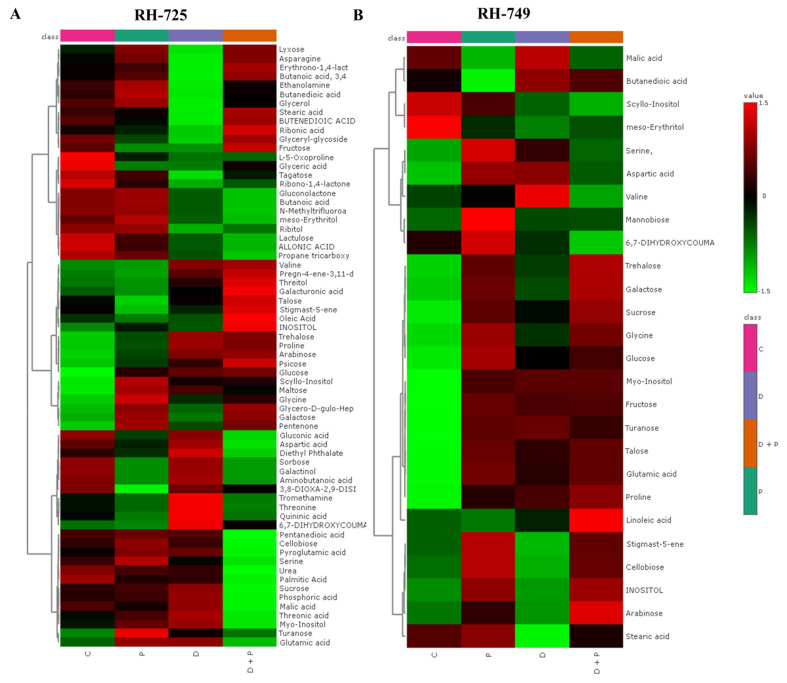
Hierarchical clustering heatmaps showing relative metabolite accumulation in leaves of *Brassica juncea* (distance measure: Pearson; Clustering algorithm: Ward), genotypes (**A**: RH-725, **B**: RH-749) under treatments (C: Control, P: PGPR, D: Drought, and D + P: Drought + PGPR). Color intensity represents the relative abundance of each metabolite: red indicates higher abundance, green indicates lower abundance.

**Figure 4:**
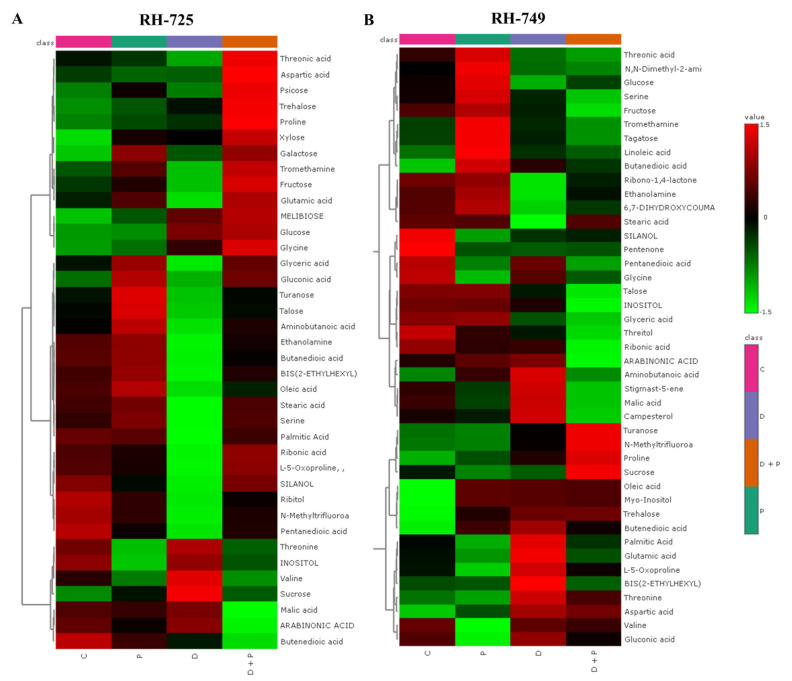
Hierarchical clustering heatmap showing the relative metabolite accumulation in roots of *Brassica juncea* (distance measure: Pearson; Clustering algorithm: Ward), genotypes (**A**: RH-725, **B**: RH-749) under treatments (C: Control, P: PGPR, D: Drought, and D + P: Drought + PGPR). Color intensity represents the relative abundance of each metabolite: red indicates higher abundance, green indicates lower abundance.

Hierarchical clustering heatmaps further revealed distinct metabolite clustering patterns across treatments, indicating significant metabolic reprogramming in response to drought and PGPR (Figure 3 and Figure 4).

In RH-725 leaves, metabolites such as proline, trehalose, glucose, and malic acid showed increased accumulation under drought and Drought + PGPR treatments. Similar patterns were observed in RH-725 roots, with elevated levels of trehalose, glucose, malic acid, and inositol under drought and further enhancement under Drought + PGPR treatment. In RH-749, fewer metabolites accumulated under drought, with notable decreases in glucose, glutamic acid, valine, and fructose in both leaves and roots. PGPR treatment led to metabolic changes in RH-749, with increases in some metabolites, but overall, there was lower accumulation than in RH-725.

### 3.3. Pathway Enrichment Analysis

MetaboAnalyst-based pathway analysis identified key metabolic pathways influenced by drought stress and PGPR treatment. Pathway enrichment analysis was performed using MetaboAnalyst. Key pathway statistics include impact score (which reflects the pathway topological score) and match status (indicating coverage as observed hits over total pathway compounds).

**Figure 5:**
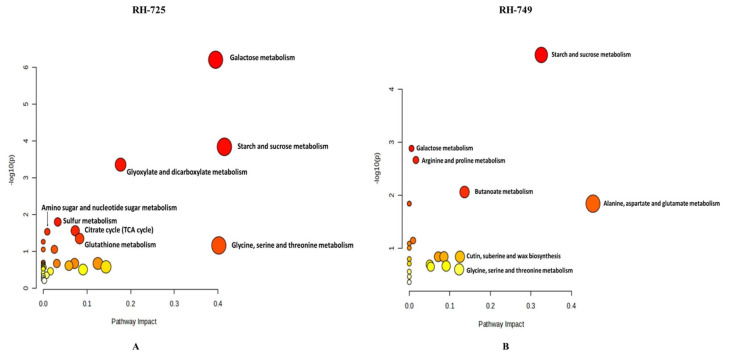
MetaboAnalyst-generated pathway enrichment plots (“metabolome view”) illustrating significantly affected metabolic pathways in response to drought stress in *Brassica juncea* genotypes (**A**: RH-725, **B**: RH-749). Arranged by the statistical significance (−log10 of *p*-value) on the y-axis and the pathway impact scores derived from pathway topology analysis on the *x*-axis. Pathway impact values refer to the cumulative percentage from the matched metabolite nodes, and the maximum importance of each pathway is 1. Circle size reflects the degree of pathway coverage (number of matched metabolites). The color of the circles represents the *p*-value, with a gradient from yellow (higher *p*-values) to red (lower *p*-values), indicating increasing statistical significance.

The effects of drought stress on metabolomic pathways in RH-725 and RH-749 have been investigated, revealing significant alterations in several key pathways. In RH-725, 35 metabolic pathways were affected, out of which only seven were significantly affected based on *p* < 0.05 and impact of more than 0. Drought stress notably impacts the galactose metabolism pathway with a high impact score. The starch and sucrose metabolism pathway also shows significant changes. Other affected pathways include glyoxylate and dicarboxylate metabolism, glutathione metabolism, sulfur metabolism, TCA metabolism, and amino sugar and nucleotide sugar metabolism (Figure 5A; Table 7).

In contrast, in RH-749, a total of 22 pathways were affected by drought stress, but only five were significantly affected. Significant changes in alanine, aspartate, and glutamate metabolism were observed with a notably high impact score. The starch and sucrose metabolism pathway also shows substantial alteration. Additional pathways affected include butanoate metabolism, arginine and proline metabolism, and galactose metabolism, each with notable impact scores (Figure 5B; Table 7). Two pathways that are affected in both genotypes are starch and sucrose metabolism, and galactose metabolism.

**Figure 6:**
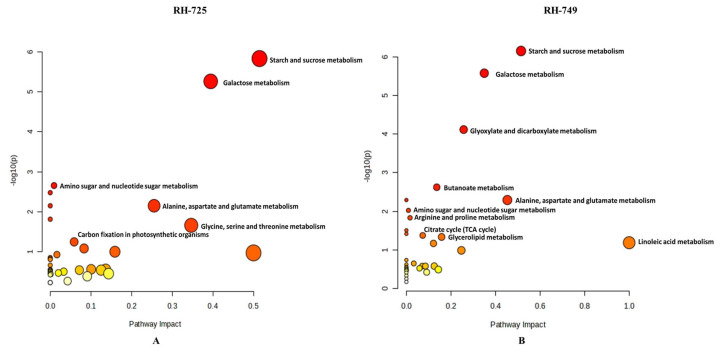
MetaboAnalyst-generated pathway enrichment plots (“metabolome view”) illustrating significantly affected metabolic pathways in response to PGPR application in *Brassica juncea* genotypes (**A**: RH-725, **B**: RH-749). Arranged by the statistical significance (−log10 of *p*-value) on the *y*-axis and the pathway impact scores derived from pathway topology analysis on the *x*-axis. Pathway impact values refer to the cumulative percentage from the matched metabolite nodes, and the maximum importance of each pathway is 1. Circle size reflects the degree of pathway coverage (number of matched metabolites). The color of the circles represents the *p*-value, with a gradient from yellow (higher *p*-values) to red (lower *p*-values), indicating increasing statistical significance.

The application of PGPR led to substantial alterations in several metabolic pathways in RH-725 and RH-749 mustard genotypes. In RH-725, 38 pathways were influenced, with 6 showing significant changes. Key pathways impacted include starch and sugar metabolism, galactose metabolism, alanine, aspartate, and glutamate metabolism. Additional pathways with significant alterations include amino sugar and nucleotide sugar metabolism, carbon fixation in photosynthetic organisms, and glycine, serine, and threonine metabolism (Figure 6A; Table 8).

Similarly, in RH-749, 34 pathways were affected, with 9 displaying significant changes. The starch and sucrose metabolism pathway exhibited the most considerable change. Other pathways with notable alterations include galactose metabolism, glyoxylate and dicarboxylate metabolism, alanine, aspartate, and glutamate metabolism, butanoate metabolism, the citrate cycle (TCA cycle), glycerolipid metabolism, arginine and proline metabolism, and amino sugar and nucleotide sugar metabolism (Figure 6B; Table 8).

## 4. Discussion

Among *Brassica* species, *B. juncea* is particularly susceptible to the adverse effects of climate change, with increasing temperatures exacerbating drought-related stress and further limiting crop performance. These environmental challenges make it crucial to implement effective strategies that enhance drought resilience and sustain crop yields [32,33]. One promising approach to mitigating drought stress is the application of Plant Growth-Promoting Rhizobacteria (PGPR). These beneficial microbes are vital in improving plant health and productivity under unfavorable conditions. PGPR functions as biofertilizers, aiding plant growth by producing phytohormones, enhancing nutrient uptake, and protecting against both abiotic and biotic stressors [34]. Research has demonstrated the positive impact of PGPR on various crops, including rice, maize, wheat, and mustard, by improving stress tolerance and overall plant performance [35,36].

Sugars such as trehalose, glucose, sucrose, talose, psicose, and xylose were significantly elevated in both roots and leaves (Table 1). These sugars contribute to osmotic regulation, ensuring cell turgor and structural integrity under drought conditions [37]. Additionally, they participate in stress signaling pathways, triggering protective responses within the plant [38]. Trehalose is related to stress response both in microorganisms and plants, having also a key role in the plant-microbe interaction [39]. The marked accumulation of sugars and sugar alcohols in RH-725 suggests heightened glycolytic activity, which subsequently fuels the tricarboxylic acid (TCA) cycle, highlighting enhanced carbon flux regulation and energy generation [40]. The increased presence of trehalose further indicates the activation of the trehalose biosynthesis pathway, which plays a crucial role in stress tolerance by stabilizing proteins and cellular membranes [41]. Similar findings have been reported by Silvente et al. [42] in soybean genotypes in response to water stress. The results of pathway analysis supported these differences between the genotypes. Trehalose is linked to the trehalose-6-phosphate (T6P) signaling pathway, which regulates sucrose homeostasis and energy balance in plants. T6P acts as a metabolic signal that modulates drought responses by influencing SnRK1 activity, thereby coordinating stress adaptation with carbon allocation.

Additionally, amino acids such as proline, glycine, and valine exhibited significant upregulation under drought stress. Proline, in particular, functions as an osmoprotectant, aiding in protein and cellular stabilization while also scavenging reactive oxygen species generated during stress conditions [43]. The accumulation of proline suggests an adaptive metabolic shift that enhances drought tolerance. Elevated amino acid levels may also contribute to energy metabolism and the synthesis of defense-related compounds [44]. The proline biosynthesis pathway, which derives proline from glutamate or ornithine, plays a crucial role in nitrogen metabolism, reflecting the plant’s ability to adjust its metabolic processes under stress [45]. Its biosynthesis via the glutamate pathway is tightly regulated under drought, reflecting an active nitrogen and redox management strategy. Glycine, a precursor in glutathione synthesis, further enhances ROS buffering capacity. The coordinated accumulation of these amino acids suggests an integrated stress response involving redox balance, osmotic adjustment, and energy redistribution [46,47].

In RH-749, although there was an increase in osmoprotectants such as proline and trehalose, their levels were considerably lower than those in RH-725, indicating a reduced capacity for osmoprotection. Similar genotype-specific responses have been reported in grapevine cultivars under water deficit, where proline levels increased by 251-fold in the drought-tolerant ‘Shiraz’ compared to 162-fold in the drought-sensitive ‘Cabernet Sauvignon’ [48]. Furthermore, the downregulation of key metabolites, including meso-erythritol, threitol, and scyllo-inositol, points to impaired metabolic pathways in RH-749 (Table 2). Metabolic pathway analysis identified seven pathways in RH-725 and five pathways in RH-749 that were significantly influenced by drought stress. Notably, RH-725 exhibited a more efficient metabolic reprogramming response to drought conditions compared to RH-749 (Table 7).

The application of Plant Growth-Promoting Rhizobacteria (PGPR) has a profound impact on the metabolite profiles of both RH-725 and RH-749, improving their stress resilience and overall growth under both normal and drought conditions. Under control conditions, PGPR treatment leads to the upregulation of key metabolites in *Brassica juncea*, particularly sugars such as galactose and trehalose, indicating an enhancement in carbohydrate metabolism [49]. Trehalose plays a critical role in plant-microbe interactions, suggesting that its accumulation may facilitate bacterial colonization by modifying carbohydrate metabolism to favour microbial establishment [50,51]. The activation of glycolysis and the tricarboxylic acid (TCA) cycle in PGPR-treated plants under optimal conditions underscores the role of these beneficial bacteria in sustaining a steady energy supply and ensuring metabolic preparedness [52]. The activation of the TCA cycle has been described as a distinctive trait for salt tolerance in Broccoli, so probably this mechanism also explains the increased tolerance to drought stress [53]. Additionally, PGPR-treated plants exhibited increased levels of metabolites involved in stress signaling and osmoprotection, such as myo-inositol, which plays a crucial role in plant defense responses [54] (Table 2). The results were consistent with Kalozoumis et al. [55], where trehalose and myo-inositol were upregulated by PGPR accumulation in tomato plants under water and nutrient stress.

Additionally, amino acids like glycine and proline are also upregulated. Elevated levels of amino acids such as proline, glycine, and valine are commonly associated with mechanisms that enhance plant resilience to drought, including regulation of stomatal aperture to control water loss, adjustment of osmotic balance to maintain cellular hydration under water stress, and protection against oxidative damage by neutralizing reactive oxygen species [56]. Extensive documentation of these amino acids’ roles in bolstering plants’ ability to withstand drought conditions can be found in studies by [46,47].

Under drought stress, the impact of PGPR becomes even more pronounced, leading to significant metabolic shifts that enhance stress resilience. PGPR-treated plants exhibit a substantial increase in metabolites directly linked to drought response mechanisms. Notably, sugars and their derivatives such as fructose, trehalose, and galacturonic acid are significantly elevated, improving the plants’ ability to regulate osmotic balance (Table 2). This osmotic regulation is essential for maintaining cell turgor and preventing dehydration under water-deficient conditions [54]. This observation was supported by prior research by Khan et al. [57] on chickpea, showing that both drought-tolerant and drought-sensitive crop genotypes can accumulate high levels of sugars when treated with PGPR, enabling them to better tolerate harsh environmental conditions.

Additionally, PGPR application promotes the accumulation of key amino acids, including proline and valine. Proline plays a vital role in drought tolerance by stabilizing cellular structures and acting as a scavenger of reactive oxygen species, thereby minimizing oxidative damage [58]. The elevated proline levels in PGPR-treated plants suggest an active proline biosynthesis pathway, reinforcing the plant’s ability to withstand drought stress [59]. Furthermore, PGPR treatment influences fatty acid metabolism, with notable increases in oleic acid and linoleic acid, which contribute to improved membrane stability [55]. Maintaining membrane integrity under drought conditions is critical for proper cellular function and for preventing the loss of essential metabolites. The enhancement of fatty acid biosynthesis in PGPR-treated plants strengthens cellular membranes, supporting stress resilience [60]. The study also revealed a metabolic shift in organic acid pathways, as evidenced by reduced levels of succinic acid, malic acid, and erythrono-1,4-lactone. This redirection of carbon flux suggests a preferential allocation of resources towards the synthesis of osmoprotectants and stress-associated amino acids (Table 3). The observed decline in malate levels in PGPR-inoculated mustard leaves may indicate its translocation from leaves to roots, facilitating bacterial colonization. Additionally, the accumulation of oleic acid in roots highlights modifications in membrane stability and signaling pathways, which are essential for plant adaptation to drought stress [61,62]. The detection of phthalates in some samples may reflect environmental or procedural contamination, as these compounds were not specifically monitored in blank runs, irrigation water, or soil. This remains a limitation of the current study and should be addressed in future metabolomic investigations. The metabolic adjustments in response to PGPR treatment were notably more pronounced in the drought-sensitive genotype RH-749 compared to RH-725. In RH-749, PGPR application under drought (D + P) significantly enhanced the abundance of osmoprotectants like proline, sugars (e.g., trehalose, galactose), and TCA cycle intermediates, indicating a robust reprogramming of primary metabolism. Pathway enrichment analysis further supported this observation, with RH-749 showing activation of diverse drought-related pathways such as glyoxylate and dicarboxylate metabolism, TCA cycle, glycerolipid metabolism, and arginine and proline metabolism, which were not prominently enriched in RH-725. These shifts suggest that PGPR application helped RH-749 mitigate stress-induced damage by enhancing energy metabolism, membrane stability, and osmotic adjustment. In contrast, RH-725, with its inherent drought tolerance, exhibited less dramatic metabolic reconfiguration, implying that PGPR support was more critical and impactful in RH-749. The limited fold change in key amino acids and sugars in RH-725 compared to RH-749 supports this interpretation. These genotype-specific and treatment-dependent patterns indicate that PGPRs contribute differently to drought tolerance mechanisms depending on the inherent stress sensitivity of the genotype. This study employed three biological replicates per treatment, which meets the standard for preliminary metabolomics. However, we acknowledge that limited replication may reduce the statistical power to detect more subtle metabolic changes. Future studies should consider increased sample sizes to improve the robustness and reliability of metabolomic analyses. This study did not include secondary metabolites or lipophilic compounds, as GC-MS is biased toward primary metabolites. Future work should utilize LC-MS or targeted metabolomics to investigate PGPR-induced changes in secondary metabolism.

## 5. Conclusions

Drought stress poses a significant challenge to *Brassica juncea* cultivation, necessitating innovative and sustainable strategies to enhance plant resilience. This study highlights the potential of a PGPR consortium consisting of *Enterobacter hormaechei, Pantoea dispersa,* and *Acinetobacter* sp. in mitigating drought-induced stress by modulating the plant’s metabolic profile. The differential accumulation of osmoprotectants, such as sugars and amino acids, and organic acids in drought-treated plants indicates enhanced osmotic balance, antioxidant defense, and energy metabolism, particularly in the drought-tolerant genotype RH-725. PGPR treatment further helps in remodelling of metabolism in both genotypes for enhanced drought stress tolerance. Metabolic pathway analysis further revealed that PGPR application influenced key biochemical pathways, including starch and sucrose metabolism, galactose metabolism, and amino acid biosynthesis, which are vital for drought adaptation. These findings suggest that PGPR can serve as an effective biological tool to improve plant survival and productivity under water-limited conditions. Notably, the effects of PGPR treatment were more pronounced in the drought-sensitive genotype RH-749, as evidenced by the activation of additional stress-responsive pathways such as the TCA cycle, glyoxylate metabolism, and arginine and proline metabolism. This suggests that PGPR had a stronger compensatory effect in RH-749, which otherwise showed a limited metabolic response under drought alone. While this study provides crucial insights into the metabolomic shifts associated with PGPR treatment, additional research is required to bridge the gap between controlled experimental conditions and real-world agricultural applications. Future studies should integrate multi-omics approaches, such as transcriptomics and proteomics, to deepen our understanding of PGPR-mediated drought tolerance at the molecular level. Furthermore, extensive field trials across different agro-climatic regions are necessary to evaluate the practical efficacy of these PGPR strains under natural stress conditions. The development of microbial consortia tailored to specific soil types and climates could enhance their effectiveness in diverse agricultural systems. Harnessing the potential of PGPR as a natural and eco-friendly approach can contribute to more sustainable cropping systems, ensuring food security and improved crop productivity in drought-prone regions.

## Figures and Tables

**Table 1:** Metabolites upregulated in response to drought stress.

Sample Name	Metabolite Name	Fold Change (Drought/ Control)	log2 (Fold Change)	Raw. *p* Val	Class
RH-725 Leaves	Trehalose	42.662	5.4149	0.004255	Sugar
	Valine	38.036	5.2493	0.023404	Amino acid
	Proline	35.548	5.1517	0.008511	Amino acid
	Glucose	9.143	3.1927	0.025532	Sugar
	Arabinose	7.8261	2.9683	0.014894	Sugar
	Diethyl Phthalate	4.3586	2.1239	0.029787	others
	6,7-DIHYDROXYCOUMARIN	4.2442	2.0855	0.021277	others
	Myo-Inositol	2.885	1.5286	0.006383	Sugar alcohol
	Tromethamine	2.8011	1.4860	0.03617	Amine
	Sucrose	2.478	1.3092	0.034043	Sugar
RH-725 Roots	MELIBIOSE	40.219	5.3298	0.006977	Sugar
	Glucose	23.863	4.5767	0.002326	Sugar
	Xylose	9.6007	3.2631	0.039535	Sugar
	Sucrose	5.1355	2.3605	0.009302	Sugar
	Valine	4.2142	2.0753	0.02093	Amino acid
	Galactose	3.5456	1.8260	0.007442	Sugar
	Glycine	3.3907	1.7616	0.013953	Amino acid
	Proline	2.9724	1.5716	0.044186	Amino acid
	Trehalose	2.5821	1.3685	0.037907	Sugar
	Malic acid	2.2786	1.1881	0.011628	Organic acid
RH-749 Leaves	Proline	24.677	4.6251	0.02963	Amino acid
	Myo-Inositol	9.0413	3.1765	0.014815	Sugar alcohol
	Talose	7.0838	2.8245	0.022222	Sugar
	Glutamic acid	3.3856	1.7594	0.048148	Amino acid
RH-749 Roots	Myo-Inositol	26.531	4.7296	0.002128	Sugar alcohol
	Trehalose	5.9953	2.5838	0.019149	Sugar
	Arabinose	5.1938	2.3768	0.03617	Sugar
	Oleic acid	5.0112	2.3252	0.017021	Fatty acid
	BIS(2-ETHYLHEXYL) PHTHALATE	4.051	2.0183	0.006383	Others
	Glutamic acid	2.9684	1.5697	0.034043	Amino acid
	Aminobutanoic acid	2.3799	1.2509	0.008511	Organic acid

**Table 3:** Metabolite upregulated in leaves in response to PGPR treatment.

Treatment	Metabolite Name	Fold Change (PGPR Treated/ Non-Treated)	log2 (Fold Change)	Raw. *p* Val	Class
RH-725 Control	Glucose	10.696	1.8823	0.012069	Sugar
	Glycero-D-gulo-Heptose	8.2461	1.6636	0.027586	Sugar
	Galactose	7.5639	1.5160	0.006897	Sugar
	Trehalose	5.6212	1.2511	0.034483	Sugar
	Glycine	5.1178	4.0542	0.017241	Amino acid
	Proline	4.8089	3.6568	0.005172	Amino acid
	Lyxose	4.321	3.0227	0.024138	Sugar
	Arabinose	3.5483	2.8626	0.005517	Sugar
	Pyroglutamic acid	3.4611	2.8371	0.022414	Amino acid
	Pentenone	3.3885	2.5677	0.018966	Ketone
	Myo-Inositol	3.3342	2.3368	0.010345	Sugar alcohol
	Turanose	3.0421	2.2903	0.003448	Sugar
	Asparagine	2.8582	1.6445	0.037936	Amino acid
	Cellobiose	2.6911	1.6114	0.036207	Sugar
	Maltose	2.3727	1.1329	0.046552	Sugar
	meso-Erythritol	2.3069	1.0086	0.032759	Sugar alcohol
	Butanoic acid	2.0422	2.9760	0.067241	Organic acid
RH-725 Drought	Fructose	43.887	2.3047	0.0075	Sugar
	Stigmast-5-ene	42.101	2.2466	0.0125	Other (Phytosterol)
	Threitol	10.74	1.9542	0.0175	Sugar alcohol
	Galacturonic acid	5.4236	1.8852	0.02	Sugar acid
	Psicose	4.9125	1.7536	0.025	Sugar
	Glycero-D-gulo-Heptose	4.5249	1.6220	0.035	Sugar
	Galactose	4.3622	1.1524	0.03	Sugar
	Oleic Acid	3.6865	1.1276	0.0325	Fatty acid
	Talose	3.168	1.8823	0.04	Sugar
	Ribonic acid	2.86	1.6636	0.005	Sugar acid
	INOSITOL	2.3803	1.5160	0.045	Sugar alcohol
RH-749 Control	Proline	16.613	1.2511	0.048276	Amino acid
	Talose	12.613	4.0542	0.010345	Sugar
	Myo-Inositol	8.1269	3.6568	0.013793	Sugar alcohol
	Mannobiose	7.2731	3.0227	0.003448	Sugar
	Trehalose	7.1458	2.8626	0.005172	Sugar
	Glutamic acid	5.9287	2.8371	0.026069	Amino acid
	Glycerol	5.0517	2.5677	0.02069	Sugar alcohol
	Glucose	4.8915	2.3368	0.031034	Sugar
	Galactose	3.1265	2.2903	0.024138	Sugar
	Maltose	3.0554	1.6445	0.018966	Sugar
	Fructose	2.193	1.6114	0.027586	Sugar
	Ribose	2.0119	1.1329	0.005862	Sugar
RH-749 Drought	Arabinose	7.8678	1.0086	0.05	Sugar
	Linoleic acid	4.9405	2.9760	0.0475	Fatty acid
	Glycero-D-gulo-Heptose	4.7457	2.3047	0.0325	Sugar
	Ribonic acid	3.875	2.2466	0.0275	Sugar acid
	Galactose	3.694	1.9542	0.0125	Sugar
	Acetin	3.3721	1.8852	0.0425	Other (Ester)
	INOSITOL	3.078	1.7536	0.01	Sugar alcohol
	Xylose	2.2229	1.6220	0.00575	Sugar
	Cellobiose	2.1849	1.1524	0.045	Sugar

**Table 4:** Metabolite downregulated in leaves in response to PGPR treatment.

Treatment	Metabolite Name	Fold Change (PGPR Treated/ Non-Treated)	log2 (Fold Change)	Raw. *p* Val	Class
RH-725 Control	Fructose	9.2746	6.2716	0.015517	Sugar
	Gluconic acid	7.612	5.2702	0.025862	Sugar acid
	Quininic acid	4.1127	3.9993	0.031034	Organic acid
	Glyceryl-glycoside	3.3472	2.4311	0.041379	Sugar
	ALLONIC ACID	2.1536	2.2174	0.005	Sugar acid
	Talose	2.0819	1.7494	0.02069	Sugar
RH-725 Drought	Quininic acid	173.71	1.4258	0.0025	Organic acid
	Sucrose	77.258	1.3987	0.005	Sugar
	Malic acid	38.591	6.7995	0.01	Organic acid
	Myo-Inositol	15.992	6.7230	0.015	Sugar alcohol
	Tromethamine	5.3932	4.2091	0.0225	Amine
	Aspartic acid	4.6507	1.6929	0.0275	Amino acid
	Threonic acid	3.3621	1.3812	0.0425	Amino acid
	6,7-DIHYDROXYCOUMARIN	2.6866	5.9800	0.0475	Others
	Threonine	2.6367	4.6330	0.0425	Amino acid
RH-749 Control	meso-Erythritol	111.39	3.1161	0.015724	Sugar alcohol
	Malic acid	105.64	2.3515	0.034483	Organic acid
	Butanedioic acid	18.495	2.2662	0.006897	Organic acid
	Scyllo-Inositol	3.2331	1.4488	0.037931	Sugar alcohol
	Linoleic acid	2.6048	1.1748	0.017241	Fatty acid
RH-749 Drought	Malic acid	63.12	1.0698	0.0075	Organic acid
	Valine	24.812	6.2716	0.0025	Amino acid
	Galacturonic acid	8.6702	5.2702	0.015	Sugar acid
	Gentiobiose	5.1037	3.9993	0.04	Sugar
	Ribono-1,4-lactone	4.8105	2.4311	0.02	Other (Lactone)
	Gluconic acid	2.7299	2.2174	0.005	Sugar acid
	Glyceric acid	2.2576	1.7494	0.03	Sugar acid
	Butanedioic acid	2.0991	1.4258	0.025	Organic acid

**Table 5:** Metabolite upregulated in roots in response to PGPR treatment.

Treatment	Metabolite Name	Fold Change (PGPR Treated/ Non-Treated)	log2 (Fold Change)	Raw. *p* Val	Class
RH-725 Control	Galactose	16.956	4.0837	0.018868	Sugar
	Uridine	11.026	3.4628	0.00566	Other (Nucleoside)
	Xylose	7.6696	2.9392	0.015094	Sugar
	Mannobiose	6.2892	2.6529	0.011321	Sugar
	Gluconic acid	4.5833	2.1964	0.001887	Sugar acid
	Talose	3.3936	1.7628	0.009434	Sugar
	Turanose	3.3881	1.7605	0.016981	Sugar
	Psicose	3.3564	1.7469	0.039623	Sugar
	Aminobutanoic acid	2.052	1.0370	0.023684	Organic acid
	MELIBIOSE	2.9273	1.5496	0.037736	Sugar
RH-725 Drought	Psicose	14.611	3.8690	0.0075	Sugar
	Proline	7.9973	3.000	0.03	Amino acid
	Galactose	3.2215	1.6877	0.0375	Sugar
	SILANOL	2.6862	1.4256	0.015	Other
	Xylose	2.427	1.2792	0.025	Sugar
	Gluconic acid	2.1935	1.1332	0.035	Sugar acid
	Lanthionine	2.1349	1.0942	0.0325	Amino acid
	Trehalose	1.7185	0.7811	0.0275	Sugar
RH-749 Control	Myo-Inositol	28.927	4.8543	0.001887	Sugar alcohol
	Tagatose	7.7416	2.9526	0.009434	Sugar
	Oleic acid	5.3344	2.4153	0.032075	Fatty acid
	Trehalose	3.5169	1.8143	0.016981	Sugar
	Threonic acid	2.2776	1.1875	0.00566	Sugar acid
	Tromethamine	2.014	1.0101	0.020755	Amine
	Ribose	2.005	1.0036	0.007547	Sugar
RH-749 Drought	Turanose	3.7283	1.8985	0.014583	Sugar
	Ribono-1,4-lactone	3.1041	1.6342	0.029167	Other (Lactone)
	Sucrose	2.9929	1.5815	0.016667	Sugar
	6,7-DIHYDROXYCOUMARIN	2.8567	1.5143	0.008333	Other
	Proline	2.7094	1.4380	0.027083	Amino acid
	Arabinose	2.091	1.0642	0.025	Sugar

**Table 6:** Metabolite downregulated in roots in response to PGPR treatment.

Treatment	Metabolite Name	Fold Change (PGPR Treated/ Non-Treated)	log2 (Fold Change)	Raw. *p* Val	Class
RH-725 Control	Tyrosine	49.825	5.6388	0.007547	Amino acid
	Threonine	9.1645	3.1961	0.003774	Amino acid
	Isoleucine	3.7534	1.9082	0.020755	Amino acid
	Valine	3.3961	1.7639	0.022642	Amino acid
	Dihydroxybutanoic acid-	3.2488	1.6999	0.035849	Organic acid
	Pentanedioic acid	2.8147	1.4930	0.033962	Organic acid
	INOSITOL	2.5141	1.3300	0.030189	Sugar alcohol
	SILANOL	2.4293	1.2805	0.013208	Other
	Propanedioic acid	2.0537	1.0382	0.050943	Organic acid
RH-725 Drought	Malic acid	390.29	8.6084	0.01	Organic acid
	Valine	25.54	4.6747	0.0225	Amino acid
	Threonine	14.752	3.8828	0.0025	Amino acid
	ARABINONIC ACID	9.0235	3.1737	0.0125	Sugar acid
	Sucrose	8.1024	3.0183	0.005	Sugar
	Butenedioic acid	4.505	2.1715	0.0175	Organic acid
	INOSITOL	4.0204	2.0073	0.02	Sugar alcohol
RH-749 Control	Gluconic acid	8.6145	3.1068	0.026415	Sugar acid
	Glycine	3.6734	1.8771	0.015094	Amino acid
	SILANOL	2.6054	1.3815	0.003774	Other
	Pentanedioic acid	2.0644	1.0457	0.030189	Organic acid
RH-749 Drought	Thymol-.beta.-d-glucopyranoside	14.275	3.8354	0.004167	Sugar
	Stearic acid	9.5412	3.2542	0.0125	Organic acid
	BIS(2-ETHYLHEXYL) PHTHALATE	3.9508	1.9821	0.002083	Other
	Ribonic acid	3.7041	1.8891	0.00625	Sugar acid
	Glutamic acid	3.5792	1.8396	0.020833	Amino acid
	ARABINONIC ACID	2.5778	1.3661	0.039583	Sugar acid
	INOSITOL	2.5033	1.3238	0.022917	Sugar alcohol
	Aminobutanoic acid	2.1473	1.1025	0.010417	Organic acid

**Table 7:** Significant metabolic pathways affected by drought stress.

Genotype	Pathway Name	Match Status (Coverage)	*p* Value	FDR	Impact
RH-725	Galactose metabolism	6/27	6.18 × 10^−07^	5.62 × 10^−05^	0.39463
	Starch and sucrose metabolism	4/22	1.44 × 10^−04^	0.0065739	0.41467
	Glyoxylate and dicarboxylate metabolism	4/29	4.42 × 10^−04^	0.013396	0.17703
	Sulfur metabolism	2/15	0.015989	0.36374	0.03315
	Citrate cycle (TCA cycle)	2/20	0.027801	0.44795	0.07318
	Amino sugar and nucleotide sugar metabolism	3/52	0.029535	0.44795	0.00927
	Glutathione metabolism	2/26	0.045338	0.58939	0.08316
RH-749	Alanine, aspartate, and glutamate metabolism	2/22	0.01453	0.18889	0.45324
	Starch and sucrose metabolism	4/22	2.28 × 10^−05^	0.002076	0.32579
	Butanoate metabolism	2/17	0.008767	0.18889	0.13636
	Arginine and proline metabolism	3/32	0.002184	0.066235	0.01637
	Galactose metabolism	3/27	0.001321	0.060087	0.00553

**Table 8:** Significant metabolic pathways affected by PGPR.

Genotype	Pathway Name	Match Status (Coverage)	*p*	FDR	Impact
RH-725	Starch and sucrose metabolism	6/22	1.46 × 10^−06^	1.33 × 10^−04^	0.51465
	Galactose metabolism	6/27	5.45 × 10^−06^	2.48 × 10^−04^	0.39463
	Glycine, serine, and threonine metabolism	3/33	0.021857	0.24862	0.34675
	Alanine, aspartate and glutamate metabolism	3/22	0.0070782	0.10735	0.2554
	Carbon fixation in photosynthetic organisms	2/21	0.046713	0.47343	0.05879
	Amino sugar and nucleotide sugar metabolism	5/52	0.0022101	0.067041	0.00927
RH-749	Starch and sucrose metabolism	6/22	7.08 × 10^−07^	6.44 × 10^−05^	0.51465
	Alanine, aspartate and glutamate metabolism	3/22	0.0051136	0.077557	0.45324
	Galactose metabolism	6/27	2.66 × 10^−06^	1.21 × 10^−04^	0.34966
	Glyoxylate and dicarboxylate metabolism	5/29	7.66 × 10^−05^	0.0023246	0.25709
	Glycerolipid metabolism	2/21	0.046154	0.35	0.15804
	Butanoate metabolism	3/17	0.0023861	0.054284	0.13636
	Citrate cycle (TCA cycle)	2/20	0.042184	0.34898	0.07318
	Arginine and proline metabolism	3/32	0.014752	0.1678	0.01637
	Amino sugar and nucleotide sugar metabolism	4/52	0.009473	0.12315	0.00927

## Data Availability

The datasets generated in this study are not publicly available but may be made available upon reasonable request to the corresponding author.

## Supplementary Materials

The following supporting information can be downloaded at: https://www.mdpi.com/article/10.3390/metabo15060416/s1. PDF S1: Chromatograms of GC-MS analysis.
