# Supplementary material for: Metabolomic Profiling Reveals PGPR-Driven Drought Tolerance in Contrasting Brassica juncea Genotypes
Source: Metabolites. 2025 Jun 19;15(6):416. doi: 10.3390/metabo15060416 (PMC12195340; doi:10.3390/metabo15060416)

Chromatograms of GC-MS analysis

1. **RH-725 Control (Leaves)**

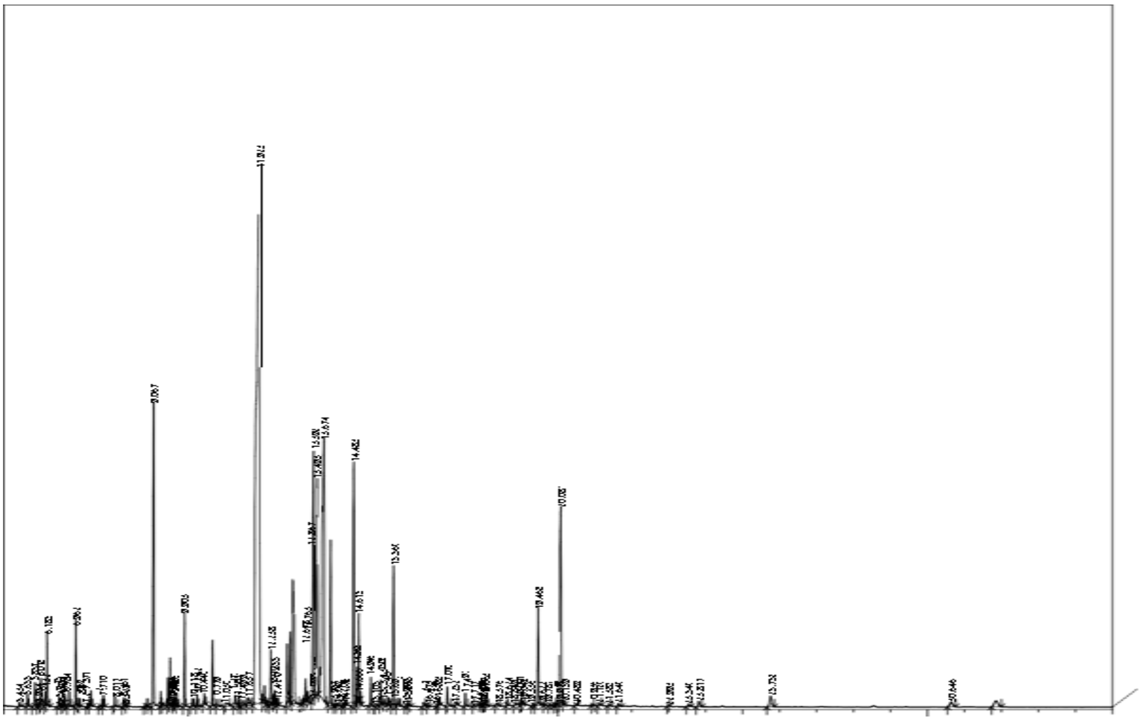

2. **RH-725 PGPR (Leaves)**

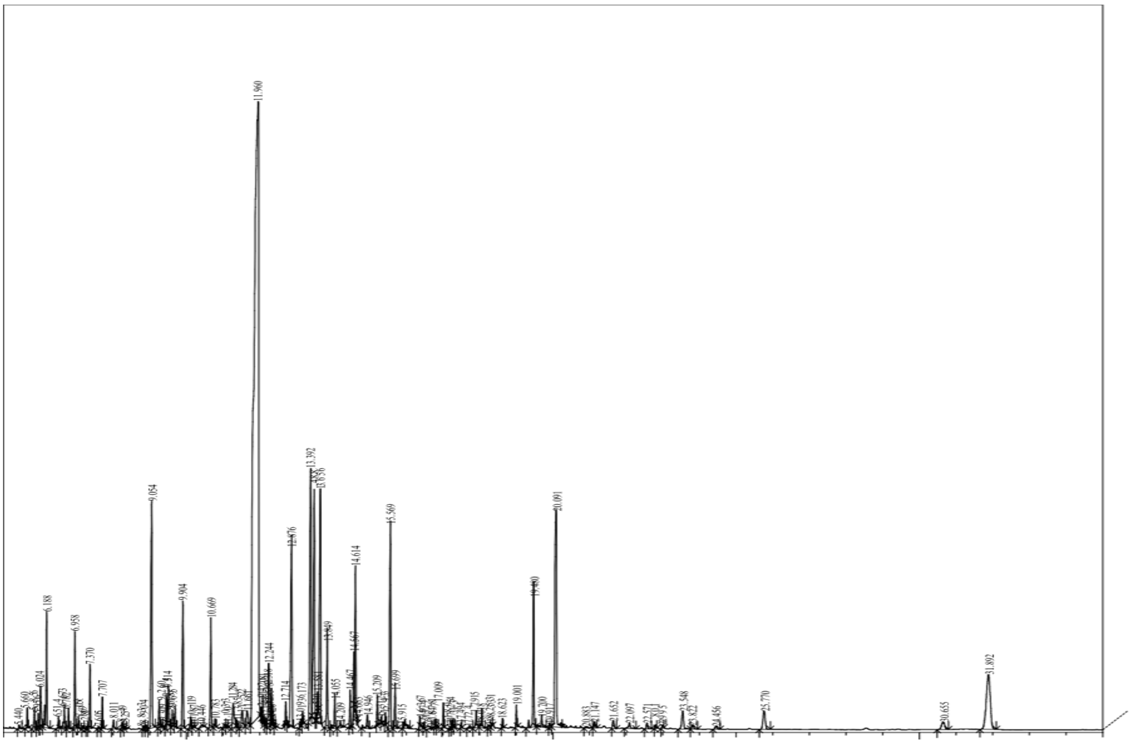

3. RH-725 Drought (Leaves)

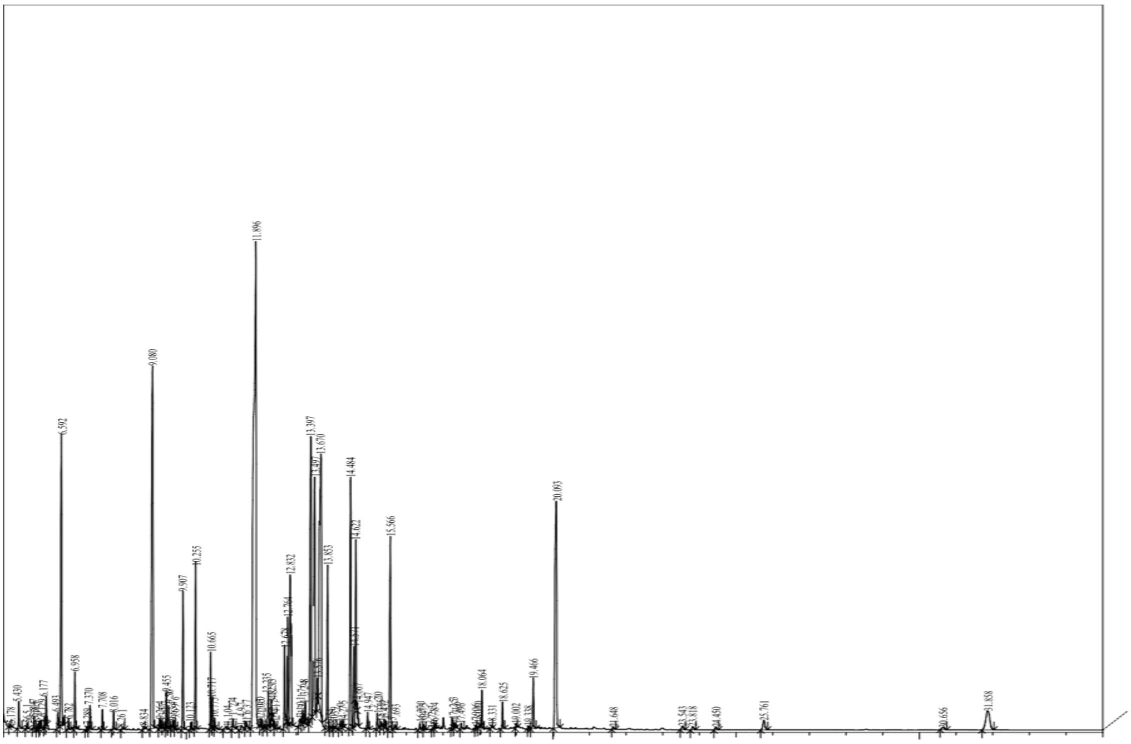

4. RH-725 Drought + PGPR (Leaves)

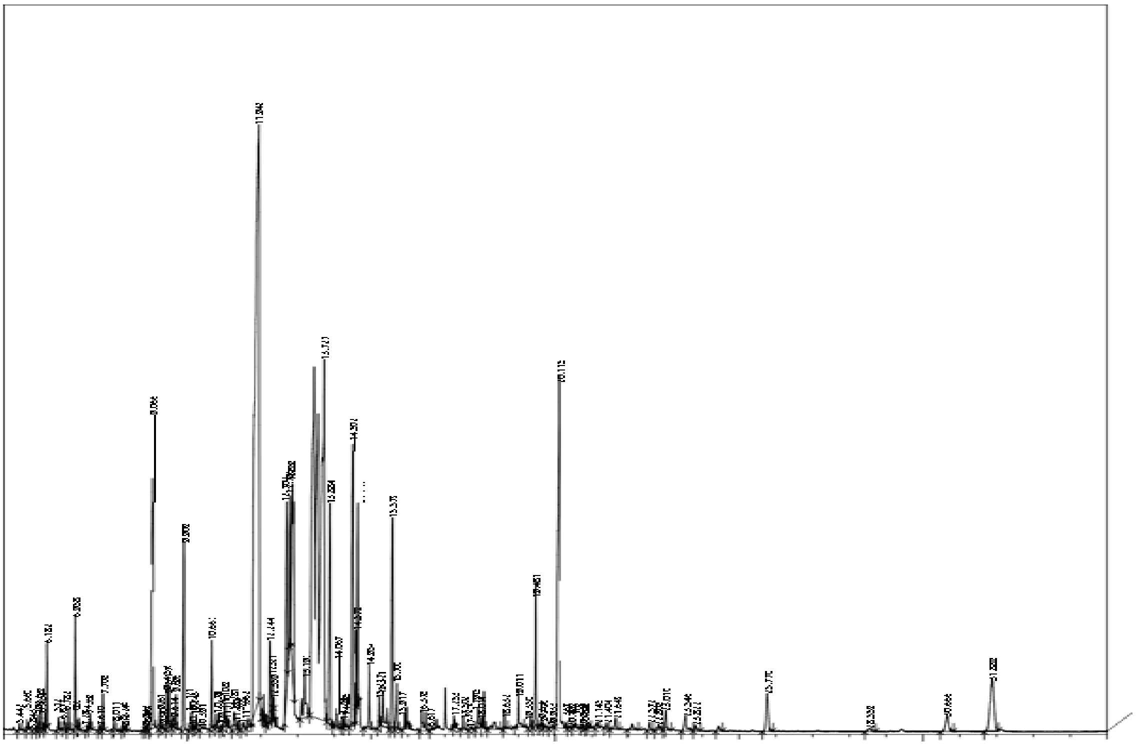

5. **RH-749 Control (Leaves)**

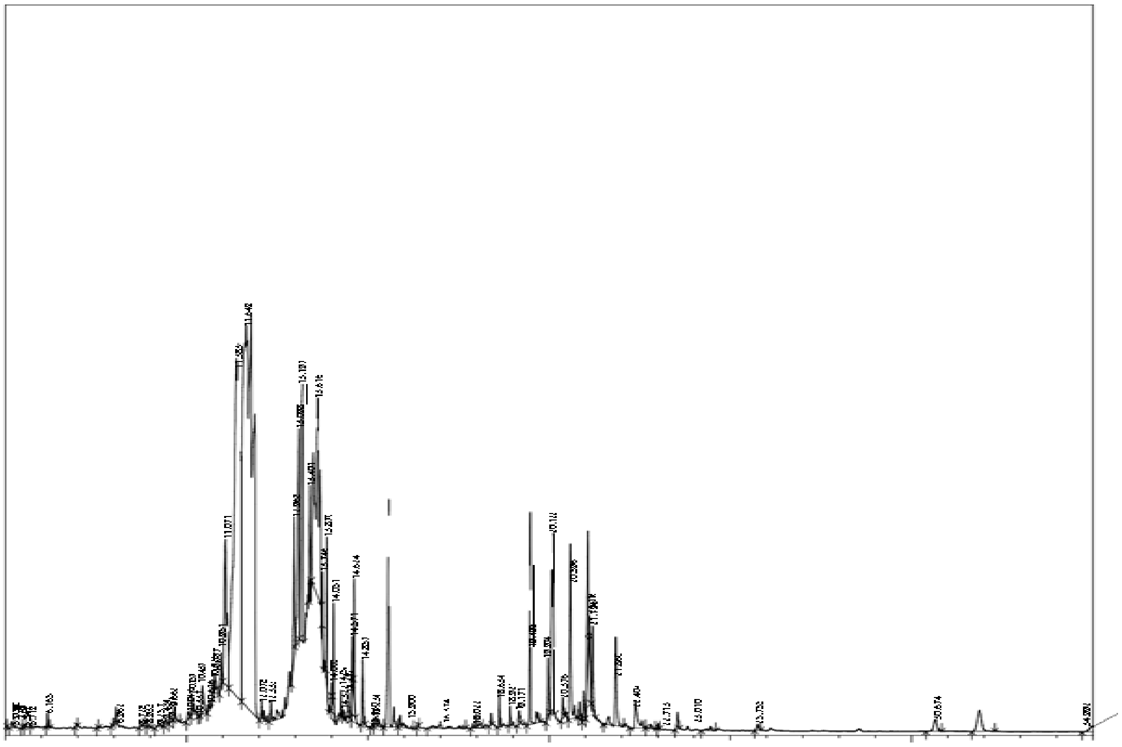

6. **RH-749 PGPR (Leaves)**

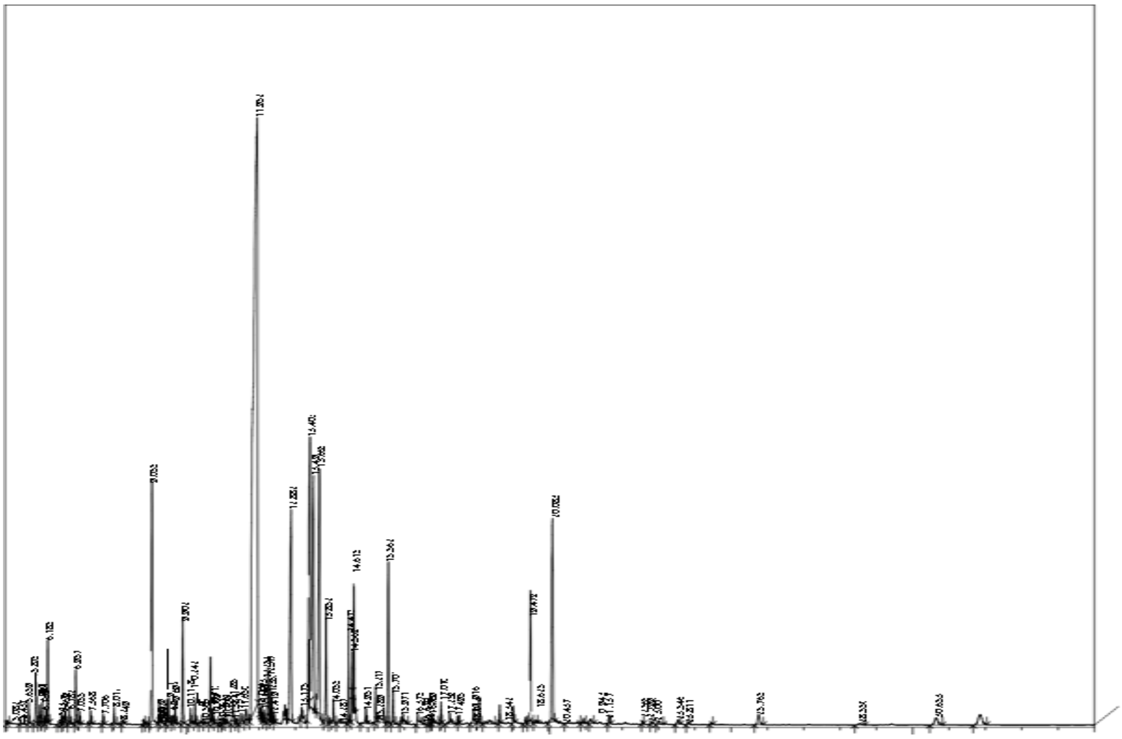

7. RH-749 Drought (Leaves)

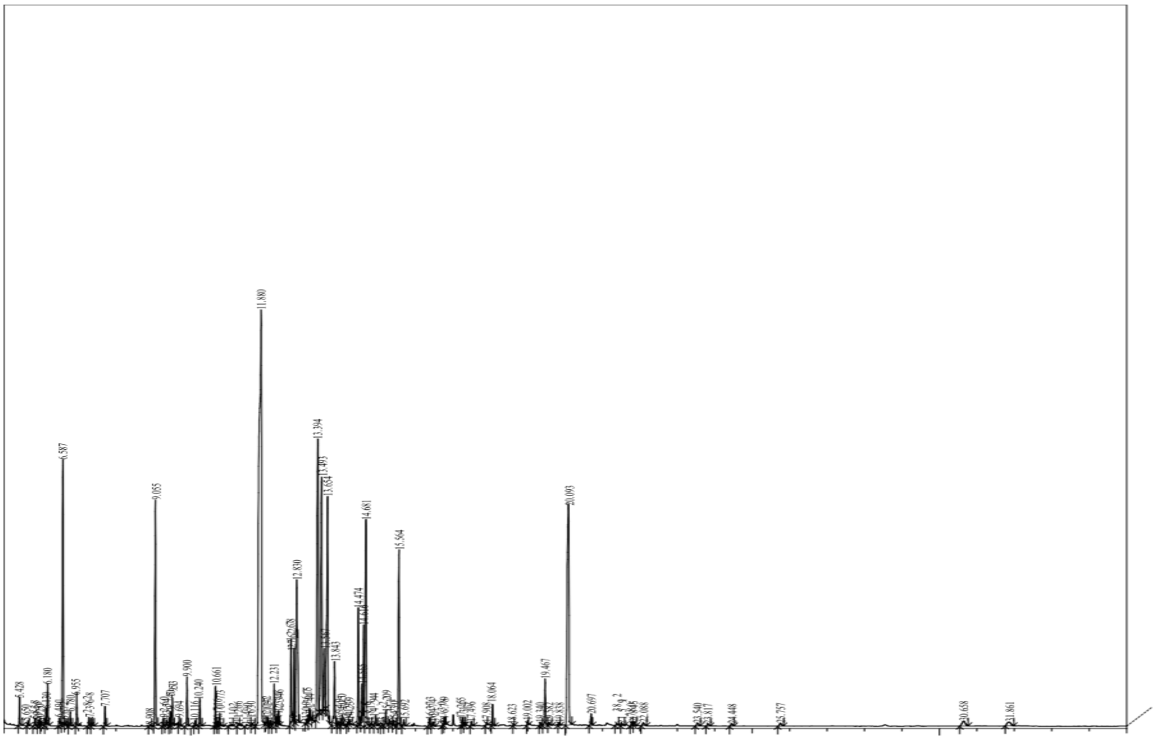

8. RH-749 Drought +PGPR (Leaves)

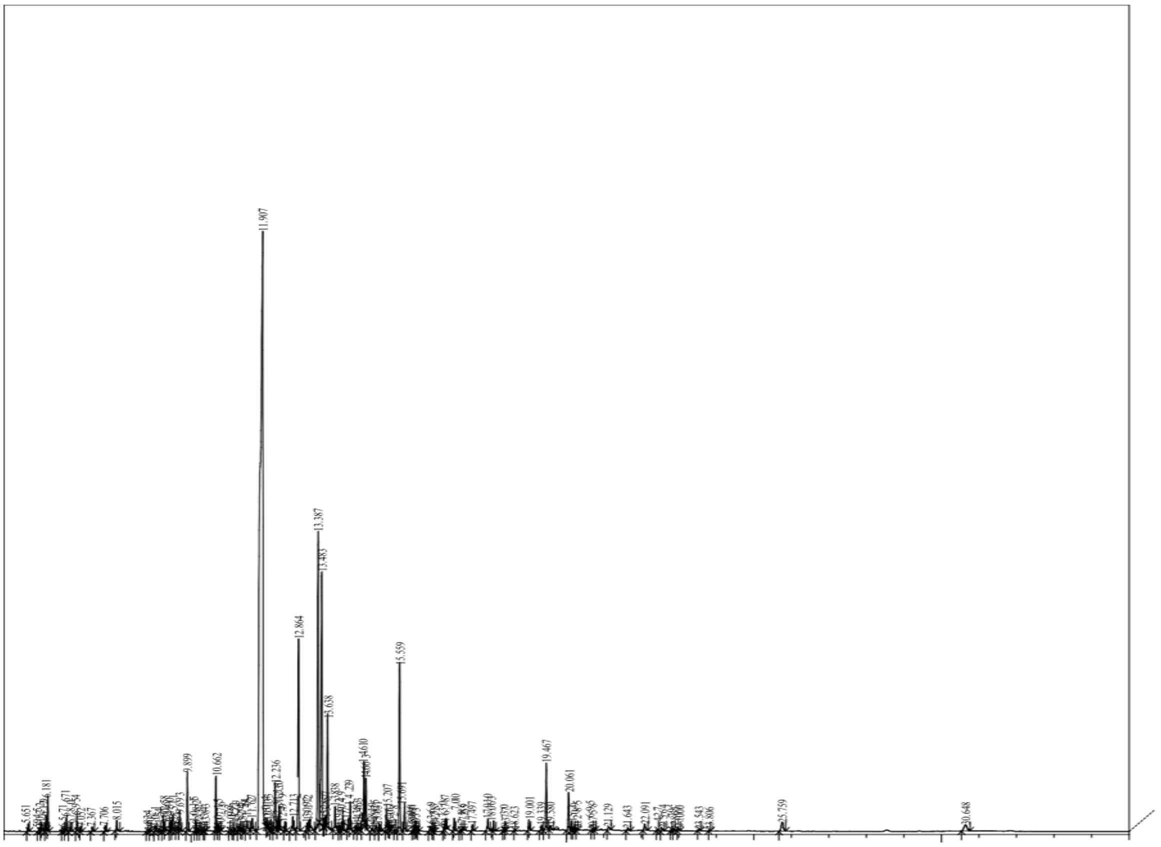

9. **RH-725 Control (Roots)**

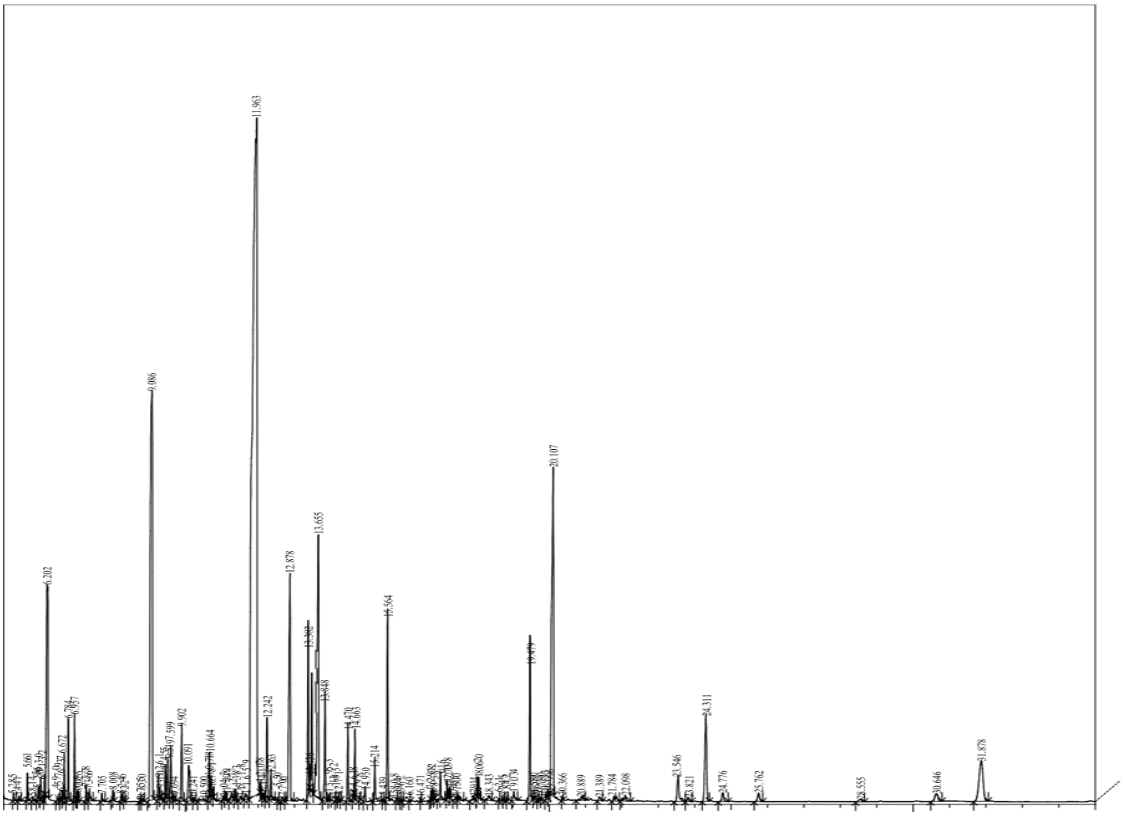

10. **RH-725 PGPR (Roots)**

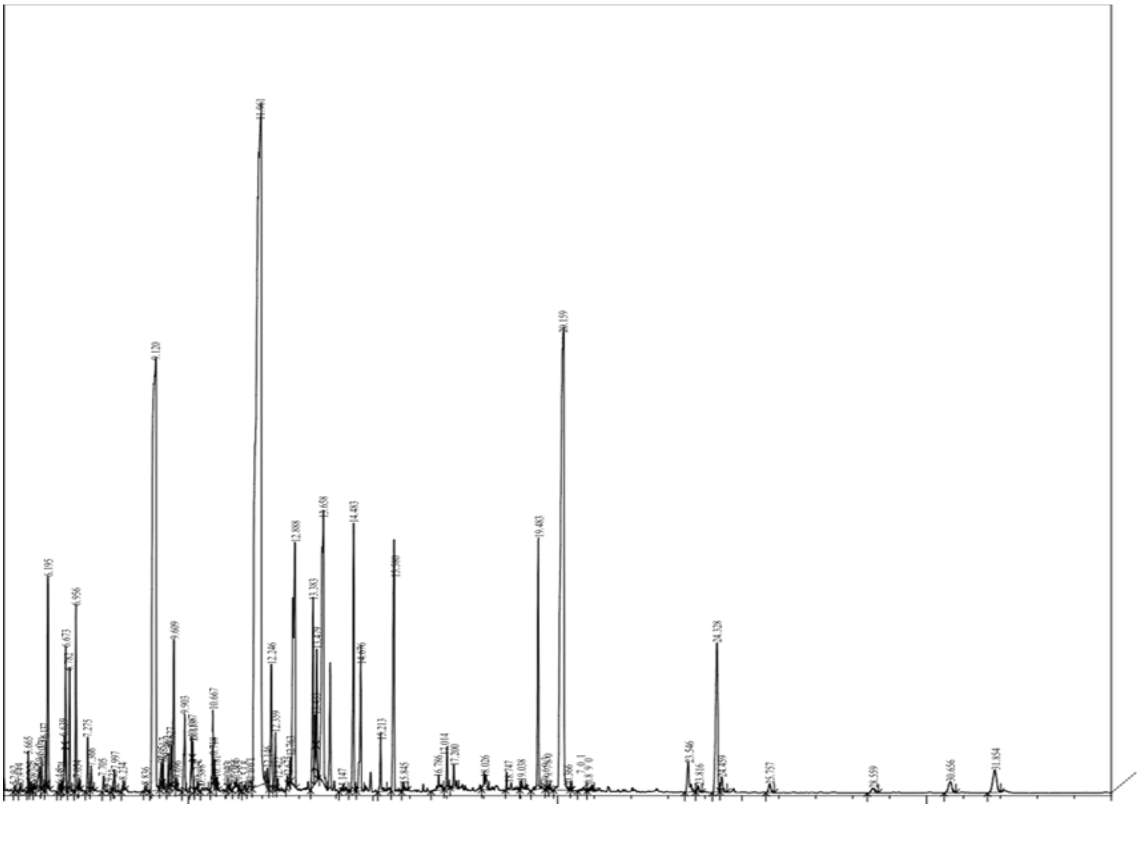

11. RH-725 Drought (Roots)

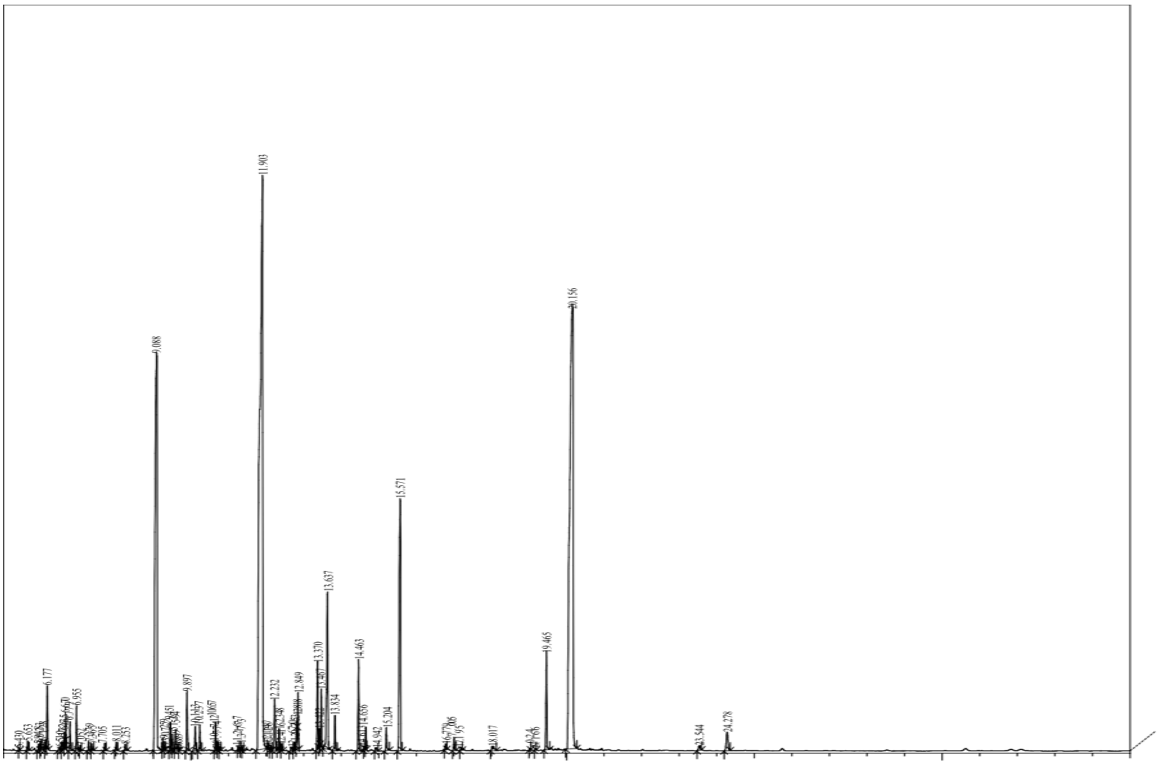

12. RH-725 Drought + PGPR (Roots)

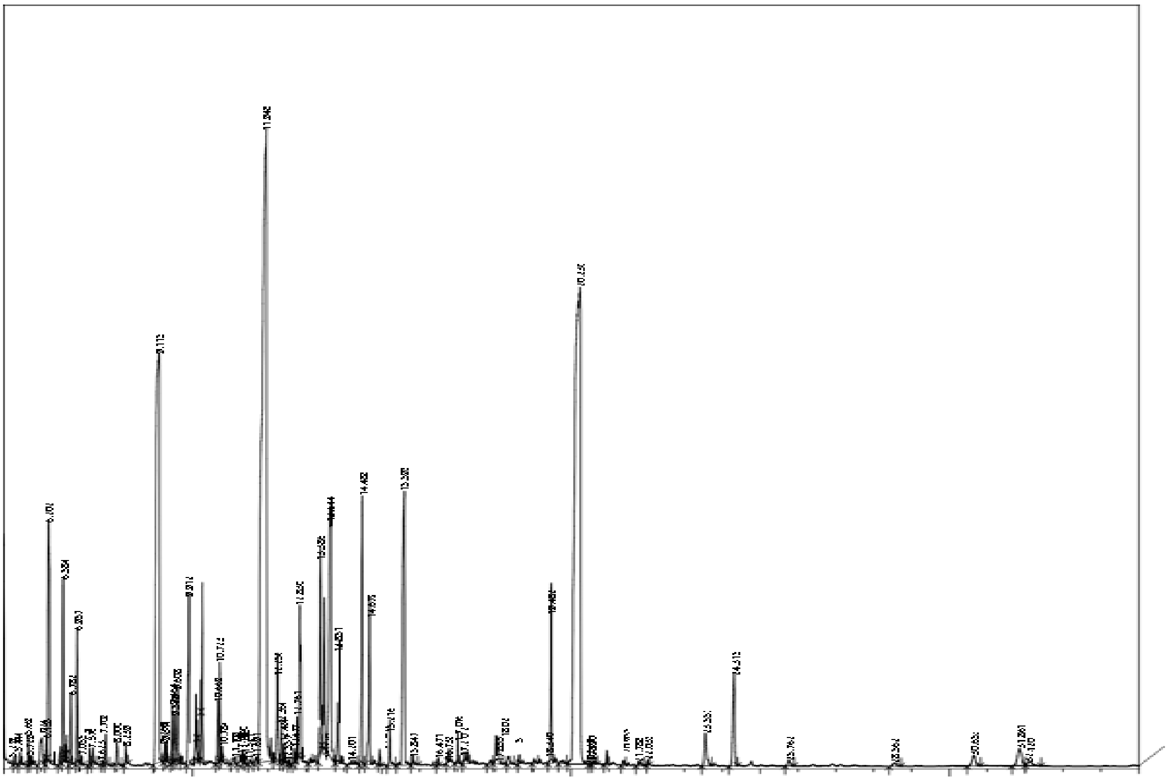

13. RH-749 Control (Roots)

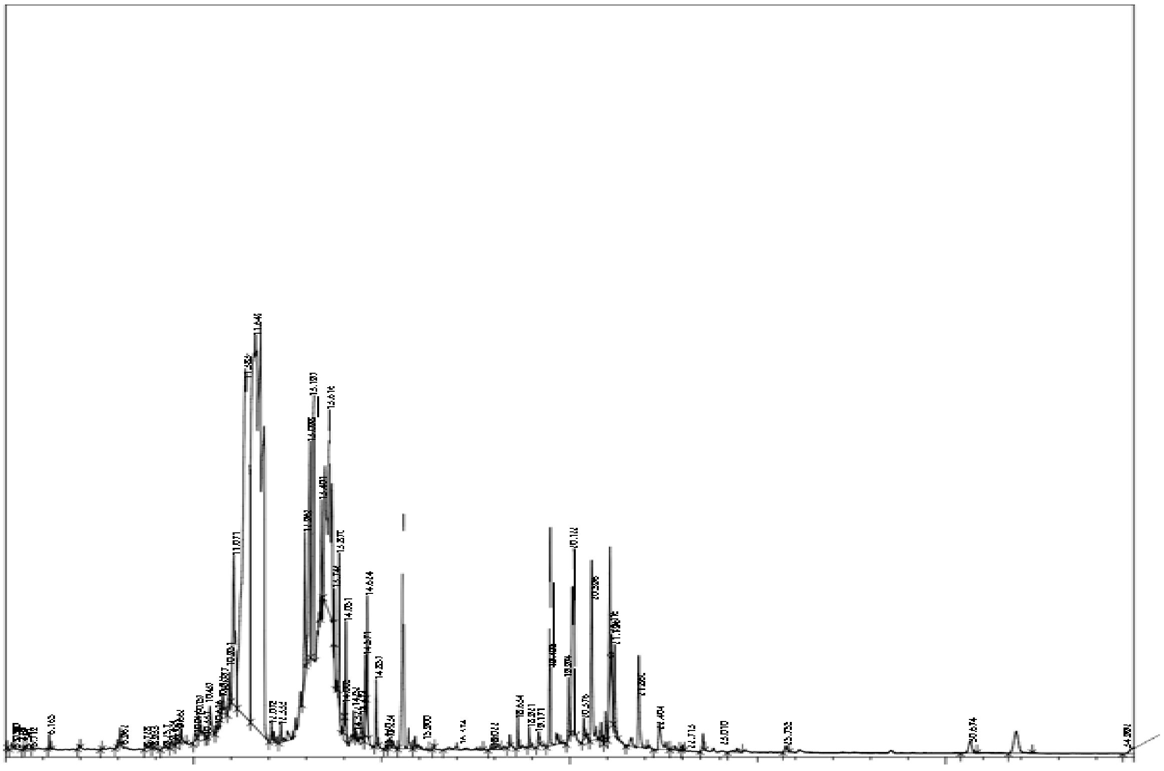

14. RH-749 PGPR (Roots)

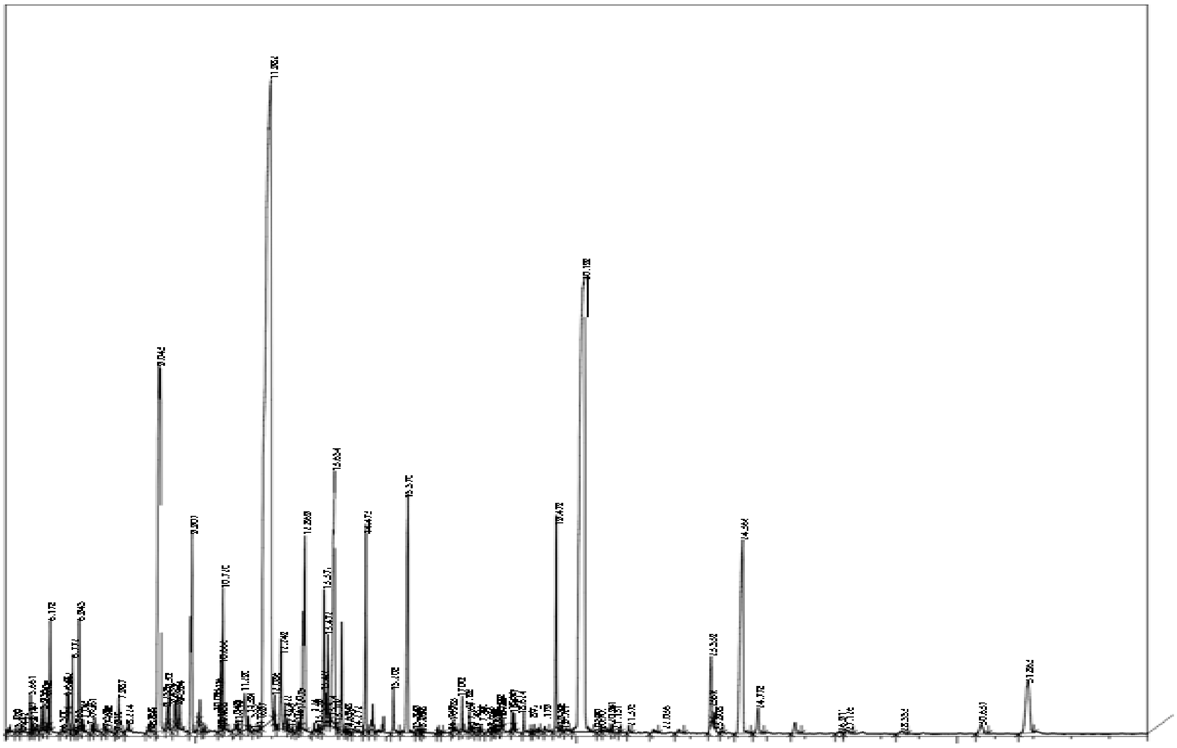

15. RH-749 Drought (Roots)

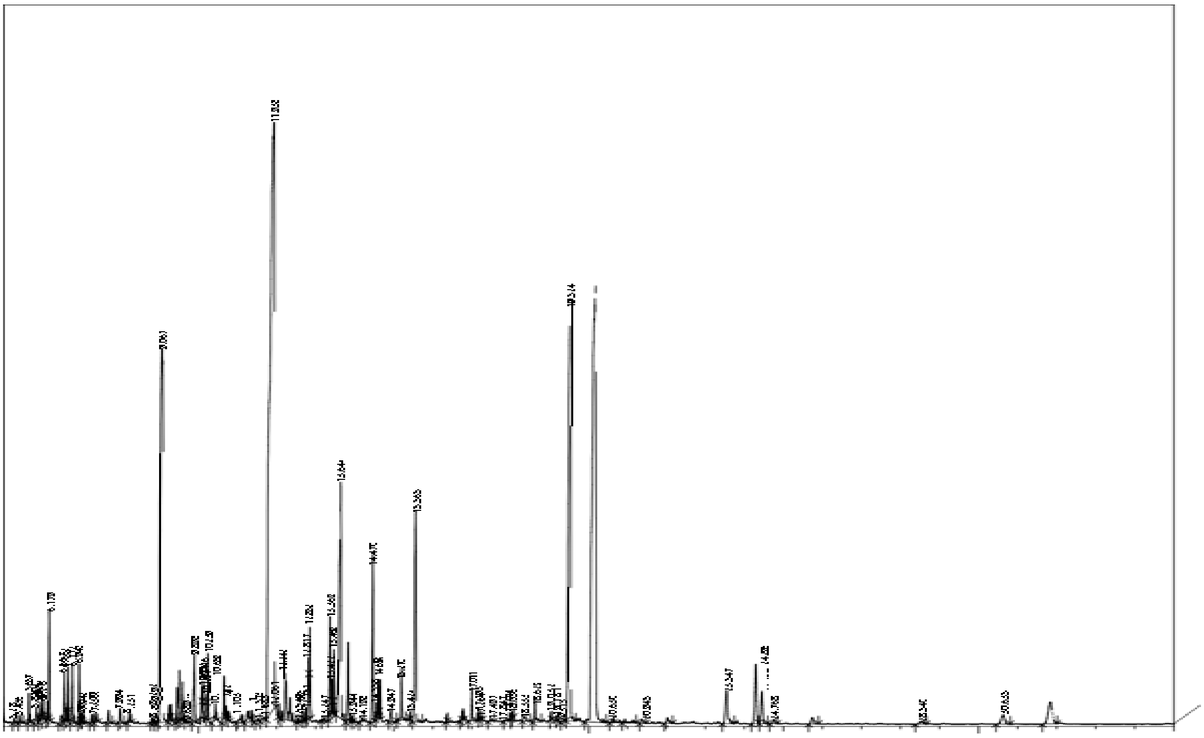

16. RH-749 Drought + PGPR (Roots)

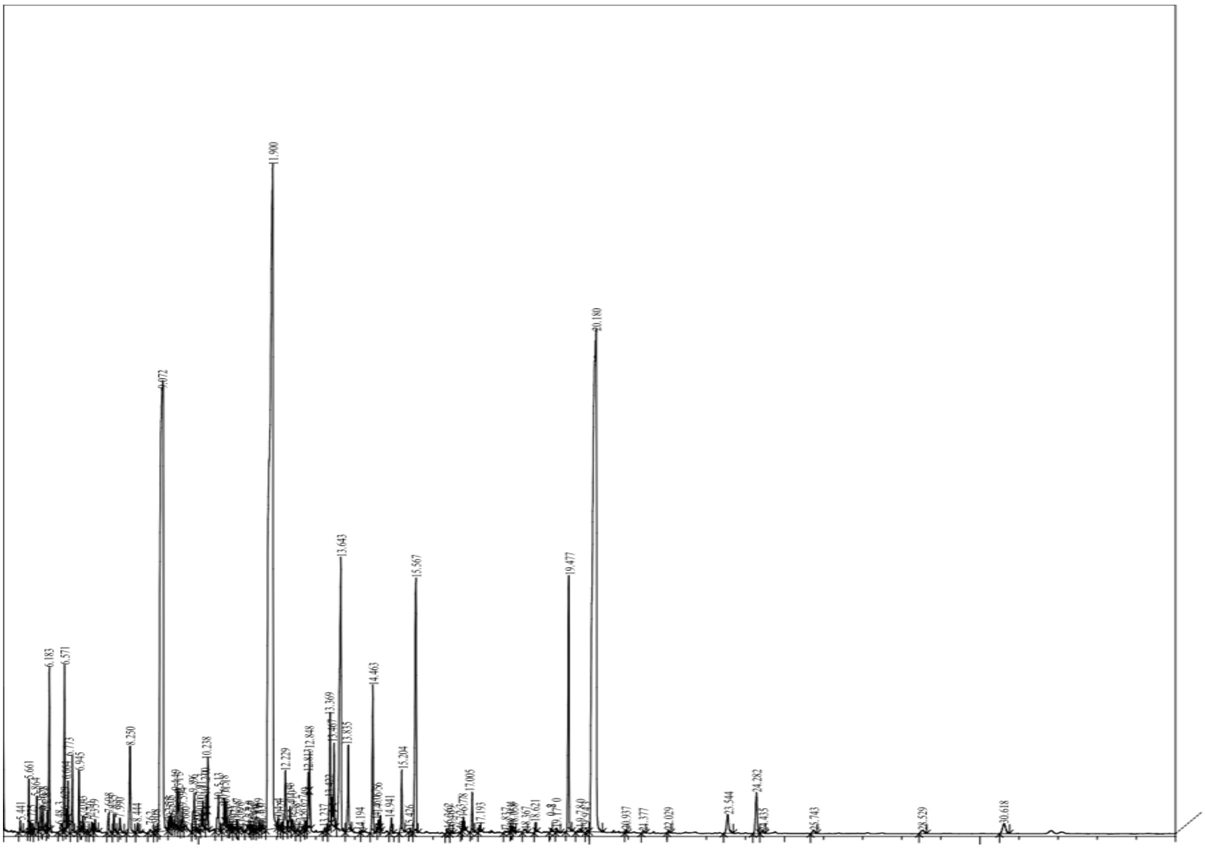

Supplement: Supplementary file 1 [file metabolites-15-00416-s001.zip › metabolites-3695341-supplementary.pdf]
